# Perception-Aware Cooperative Path Planning for Multi-UAV Systems in Urban Wind Fields via Deep Reinforcement Learning

**DOI:** 10.3390/s26102960

**Published:** 2026-05-08

**Authors:** Jie Ding, Linshen Wang, Shuxin Jin, Di Wang

**Affiliations:** 1 School of Civil Engineering and Architecture, University of Jinan, Jinan 250022, China; dingjie@stu.ujn.edu.cn (J.D.); cea_wangls@ujn.edu.cn (L.W.); 2 School of Intelligent Systems Engineering, Sun Yat-sen University, Shenzhen 518107, China; jinshx3@mail.sysu.edu.cn; 3 Department of Civil and Environmental Engineering, University of Auckland, Auckland 1023, New Zealand

**Keywords:** Unmanned Aerial Vehicles (UAVs), environmental perception, sensor-driven navigation, deep reinforcement learning, cooperative path planning, wind field disturbances

## Abstract

The safe deployment of multiple Unmanned Aerial Vehicles (UAVs) in complex urban environments relies heavily on accurate environmental perception and efficient cooperative path planning. However, executing multi-UAV operations in low-altitude airspaces faces severe challenges due to the dual constraints of complex building clusters and steady-state wind field disturbances. These dynamic environmental factors frequently distort sensory expectations, inducing trajectory drift and degrading policy robustness. To address these limitations, this paper proposes an enhanced Dueling Double Deep Q-Network (D3QN) algorithm, termed NPD3QN, tailored for perception-aware multi-UAV cooperative path planning. By formulating the perceived environmental data (e.g., wind speed, obstacle distances, and inter-UAV states) into a Markov Decision Process, an *N*-step update strategy is integrated to enhance the characterization of long-term returns. Simultaneously, an improved Prioritized Experience Replay (PER) mechanism is developed to actively filter negative experiences and assign dynamic weights to critical state-action samples, thereby significantly elevating training stability. A 3D urban kinematic environment incorporating a steady-state simulated wind field is constructed. Extensive ablation and comparative results demonstrate that NPD3QN effectively maps high-dimensional state perceptions to robust control commands. In wind-disturbed scenarios, it generates highly streamlined cooperative trajectories, reducing the total path length by approximately 11.7% compared to the standard D3QN baseline. While currently evaluated within steady-state simulated constraints, this study establishes a robust, sensor-driven methodological foundation for autonomous multi-UAV cooperative path planning in wind-disturbed airspaces.

## 1. Introduction

In recent years, the rapid advancement of intelligent autonomous systems in sensing, computation, and decision-making has catalyzed the integration of Unmanned Aerial Vehicles (UAVs) into various civilian applications [1]. Driven by the burgeoning requirements of Urban Air Mobility (UAM), UAVs are increasingly deployed for low-altitude logistics delivery, emergency medical rescue, real-time traffic monitoring, and critical infrastructure inspection [2]. Within these contexts, the urban low-altitude airspace has emerged as a vital spatial medium for complex mission execution. A fundamental challenge in such operations is enabling UAVs to achieve autonomous obstacle avoidance and precise target reaching within unfamiliar and constrained environments. This capability hinges on autonomous decision-making to navigate around obstacles and identify optimal trajectories—a challenge formulated essentially as a complex path planning problem [3]. In this context, cooperative navigation serves as the overarching sensor-driven framework, while path planning acts as its core decision-making and execution mechanism. Effective path planning is not only critical for ensuring the operational safety of UAV fleets but also serves as a primary determinant of mission efficiency. Consequently, developing efficient and reliable path planning methodologies for complex urban low-altitude environments is of paramount scientific and practical significance. To achieve this, modern UAVs heavily rely on diverse onboard sensors (e.g., LiDAR, IMU, and anemometers) to acquire real-time environmental states. The efficient processing and fusion of these multi-modal sensory inputs into safe navigational commands remain a core challenge in autonomous system design.

Compared to ground-based path planning, low-altitude path planning offers enhanced 3D spatiality and maneuverability; however, urban environments introduce significantly more rigorous constraints [4]. First, the high density and structural complexity of building clusters severely restrict the traversable airspace, necessitating obstacle avoidance within extremely limited safety margins. Second, local wind fields are highly susceptible to urban topography and building layouts, where significant variations in wind speed and direction can induce path deviations and increase flight distance, even escalating to collision risks. Therefore, UAV path planning in urban airspace must explicitly account for wind field disturbances to ensure the flyability and safety of the generated trajectories [5]. Furthermore, urban missions typically involve multi-UAV systems operating in shared airspace. Beyond individual obstacle avoidance, these systems require sophisticated spatiotemporal coordination to resolve conflicts while maintaining global operational efficiency—for instance, by minimizing redundant detours and reducing path crossing risks. When subjected to steady-state wind disturbances, the resulting path drift further complicates the relative motion dynamics between drones [6], making it increasingly difficult to avoid collisions and coordinate maneuvers. Thus, multi-UAV collaborative path planning that incorporates both structural building constraints and steady-state wind-field disturbances represents a high-value research frontier.

Current methodologies for solving path planning problems are generally categorized into three paradigms: traditional algorithms, intelligent metaheuristic algorithms, and deep reinforcement learning (DRL) [7]. Traditional algorithms typically assume static and interference-free environments [8]; while performant in simple or well-mapped scenarios, they exhibit significant limitations in complex urban settings. Specifically, when facing external perturbations such as wind-field disturbances, traditional methods often lack the requisite adaptability and real-time responsiveness for practical deployment. Intelligent metaheuristic (bionic) algorithms, characterized by their simulation of natural swarming behaviors and local search mechanisms, offer a viable pathway for trajectory optimization under multi-objective and multi-constraint conditions [9]. However, these approaches rely heavily on iterative stochastic optimization, which often leads to prohibitive computational overhead, slow convergence in high-dimensional state spaces, and a tendency to become trapped in local optima. Such bottlenecks fundamentally limit the efficiency and precision of path planning in time-sensitive urban scenarios.

The integration of Deep Reinforcement Learning (DRL) into sensor-based path planning represents a paradigm shift from traditional bio-inspired algorithms. Unlike heuristic-based approaches that rely on pre-defined search strategies, DRL agents learn optimal decision-making policies through continuous interaction with the environment, effectively mapping high-dimensional raw sensory inputs directly to continuous or discrete control actions [10]. This inherent adaptability is particularly advantageous for Urban Air Mobility (UAM), where UAVs must navigate steady-state low-altitude wind fields and complex urban geometries with high flexibility.

Recent advancements have established DRL as a cornerstone of autonomous navigation research. For instance, Tang et al. developed an enhanced Dueling Double Deep Q-Network (D3QN) framework utilizing Prioritized Experience Replay (PER) for UAV path planning in dynamic scenes, demonstrating significant performance gains over classical methods [11]. Similarly, Farid et al. integrated PER with $L_{2}$-regularization within a Deep Q-Network (DQN) architecture to improve the success rate of autonomous UAV agents in 3D obstacle-cluttered environments, addressing the limitations of baseline DQN models in high-dimensional state spaces [12]. The efficacy of DRL extends to robust obstacle avoidance and path planning in multi-objective scenarios. Traditional analytical methods often struggle with complex control laws, a challenge overcome by the DQN-based collision avoidance algorithms [13]. For quadrotor UAVs, autonomous obstacle avoidance can be achieved through two-stage architectures comprising perception and decision modules, even in the absence of prior environmental information [14]. To balance efficiency and collision risk, globally guided reinforcement learning methods combine global path planning with DRL-based local planners, utilizing novel reward structures that generalize across diverse dynamic environments [15]. Performance in these dynamic settings can be further optimized by combining D3QN with PER to enhance sampling efficiency and value estimation stability, while Gök demonstrated that this combination significantly reduces path length and travel time for mobile robots without compromising safety [16].

Advanced architectural modifications have further addressed the constraints of partially observable and continuous dynamic environments. For mobile robots in unknown settings, reward-modified Double DQN (DDQN) algorithms utilize weighted reward functions and multi-level thresholds to stabilize the training process against erratic reward signals caused by moving obstacles [17]. In maritime contexts, integrating Long Short-Term Memory (LSTM) networks with DRL enables drones to mitigate decision biases stemming from partial observability by preserving long-term temporal dependencies [18]. Efficient exploration and faster convergence are also facilitated by incorporating prior knowledge and rule-based constraints into the DQN framework [19]. Furthermore, modern hybrid approaches have successfully addressed complex mission requirements. Liu et al. proposed an enhanced DRL strategy for UAVs in complex dynamic environments that incorporates a novel data storage system and an artificial potential field (APF) strategy to maintain high mission success rates [20]. These innovations collectively enable UAVs to navigate high-density dynamic obstacles while maintaining path optimality and mission reliability.

Despite the theoretical advancements in UAV trajectory optimization, multi-UAV cooperative navigation in complex urban airspaces remains an evolving research frontier. Recent paradigms have begun to bridge game theory with multi-agent systems (MASs) to model strategic interactions and robust path optimization under high-dimensional uncertainties [21]. To address the real-time constraints and network complexity in large-scale swarms, autonomous connection selection mechanisms based on network formation games have been proposed to optimize positioning accuracy and computational efficiency [22]. Furthermore, hierarchical frameworks integrating LSTM-based dynamic predictors and multi-source consistency evaluation (e.g., wind-speed and airflow data) have demonstrated enhanced formation stability in challenging or GNSS-denied environments [23]. While these efforts have significantly improved cooperative reliability, current research specifically addressing multi-UAV operations under steady-state wind-field disturbances in dense urban canyons remains relatively sparse. Existing frameworks often overlook the tight kinematic coupling between 3D obstacle avoidance and multi-agent safety constraints when subjected to environmental drift.

To address these limitations, this paper proposes NPD3QN, an architecture tailored for robust multi-UAV coordination in wind-disturbed urban airspaces. While the core algorithmic components—D3QN, *N*-step targets, and PER—are established reinforcement learning techniques, the main contributions of this work are summarized as follows:

- **Kinematic-Aware Decision Framework:** We integrate domain-specific kinematic wind field coupling into the state transition and reward design. This multi-objective reward explicitly incentivizes tailwind utilization and crosswind rejection, enabling robust navigation that standard DRL frameworks fail to capture.
- **Task-Oriented Experience Replay:** We propose an improved PER mechanism that regulates sampling density based on wind-induced drift patterns and collision outcomes. This mitigates conservative policy freezing and ensures safe exploration in high-variance environments.
- **Enhanced Cooperative Trajectory Optimality:** By synergizing the *N*-step update strategy with kinematic perception, the proposed NPD3QN effectively maps high-dimensional sensor data to streamlined, collision-free maneuvers, significantly reducing redundant detours under complex multi-agent constraints.

To contextualize our contributions within the evolving landscape of UAV navigation, a nuanced comparative summary of related works is provided in Table 1. Recent studies have made significant strides in optimizing single-agent obstacle avoidance, enhancing sampling efficiency, and addressing foundational multi-agent coordination. Building upon these robust methodological foundations, our work specifically investigates the localized synergy between multi-agent IQL coordination and kinematic-aware reward structures under modeled steady-state wind disturbances.

## 2. Methodology

### 2.1. Problem Formulation

The decision-making process for UAV path planning is formulated as a Markov Decision Process (MDP), which provides a rigorous mathematical framework for modeling sequential decision problems under uncertainty. Formally, the MDP is characterized by a tuple 〈S,A,P,R,γ〉. Here, *S* denotes the state space, representing the set of all possible configurations the UAV may occupy within the operational environment. *A* signifies the action space, encompassing the set of all executable maneuvers available to the UAV. The reward function $R(s_{t},a_{t},s_{t+1})$ quantifies the immediate feedback received upon executing action $a_{t}$ in state $s_{t}$ and transitioning to a subsequent state $s_{t+1}$. The transition probability $P(s_{t+1}|s_{t},a_{t})$ defines the likelihood of shifting to state $s_{t+1}$ given the current state $s_{t}$ and action $a_{t}$. Finally, γ∈[0,1] is the discount factor, which determines the present value of future rewards and governs the agent’s horizon of optimization.

In the context of path planning, the agent interacts with the environment to acquire the necessary information to solve the MDP, a process facilitated through reinforcement learning (RL). The primary objective of RL is to derive an optimal policy that maximizes the cumulative reward, ensuring that the agent consistently selects the action that maximizes the *Q*-value. The optimal *Q*-value function is expressed via the Bellman equation:

(1) $$Q^{*}\left(s_{t},a_{t}\right)=E\left[R\left(s_{t},a_{t},s_{t+1}\right)+\gamma \max_{a_{t+1}}Q^{*}\left(s_{t+1},a_{t+1}\right)\right]$$

where $a_{t+1}$ represents the potential action to be taken in the subsequent state $s_{t+1}$ to maximize the expected future value. By leveraging this framework, the agent evaluates the utility of various paths by synthesizing environmental data with its own internal state, thereby guiding the UAV to make informed and autonomous path planning decisions in complex scenarios.

### 2.2. Kinematic Environment and Wind Field Modeling

In UAV path planning, the wind field is a critical environmental factor that significantly influences flight efficiency and operational safety. While tailwinds can minimize energy consumption and enhance operational efficiency, head-winds and cross-winds often induce trajectory drift, potentially compromising obstacle avoidance and the quality of multi-UAV collaborative path planning. To ensure that the learned path planning strategies remain executable and robust under atmospheric disturbances, this study incorporates a wind field into the RL training environment. The wind field is integrated as a core component of the environmental dynamics and reward feedback, enabling the agent to perform adaptive decision-making based on meteorological conditions.

The wind field is modeled as a steady-state spatial vector field in three-dimensional space, defined as:

(2) $$V_{wind}\left(x,y,z\right)={\left[U\left(x,y,z\right),V\left(x,y,z\right),W\left(x,y,z\right)\right]}^{T}$$

where *U*, *V*, and *W* represent the wind velocity components along the three principal axes, respectively. Within the RL framework, wind field information is embedded into multiple stages of the decision-making pipeline:

- **State Perception**: Wind conditions are incorporated into the state vector, allowing the agent to directly perceive the wind direction and magnitude at its current coordinates.
- **State Transition**: The environment accounts for geometric offsets by superimposing the wind-induced drift onto the intentional displacement generated by discrete actions, thereby simulating realistic aerodynamic effects on the UAV trajectory.
- **Reward Design**: Directional feedback related to the wind field is introduced. This encourages the policy to satisfy obstacle avoidance and goal-reaching constraints while favoring advantageous wind directions and suppressing ineffective yaw caused by cross-winds, leading to more efficient and stable path generation under UAM constraints.

**Figure 1:**
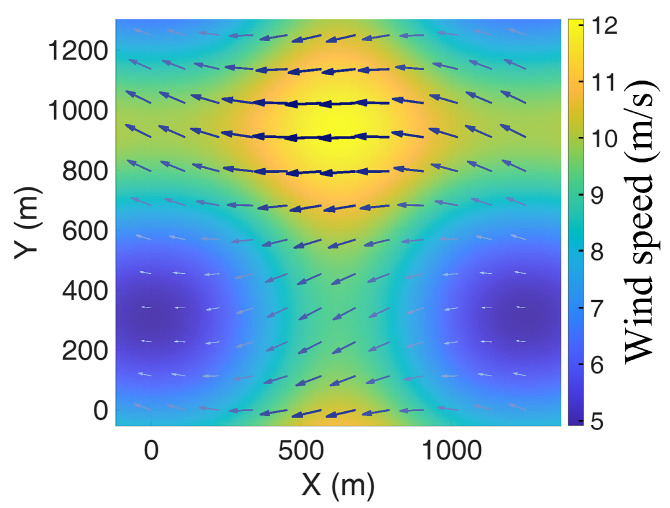
Two-dimensional wind field visualization. Arrows indicate wind direction, and colors represent wind speed magnitude.

**Figure 2:**
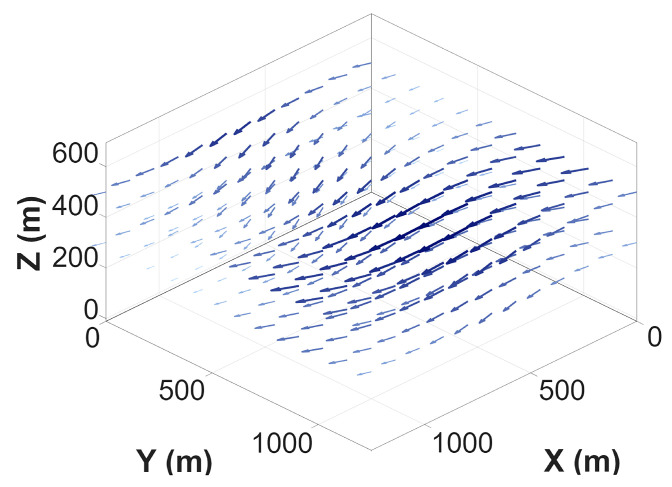
Three-dimensional isolated wind field visualization without complex urban terrain. Arrows indicate wind direction, and arrow size indicates wind speed.

**Figure 3:**
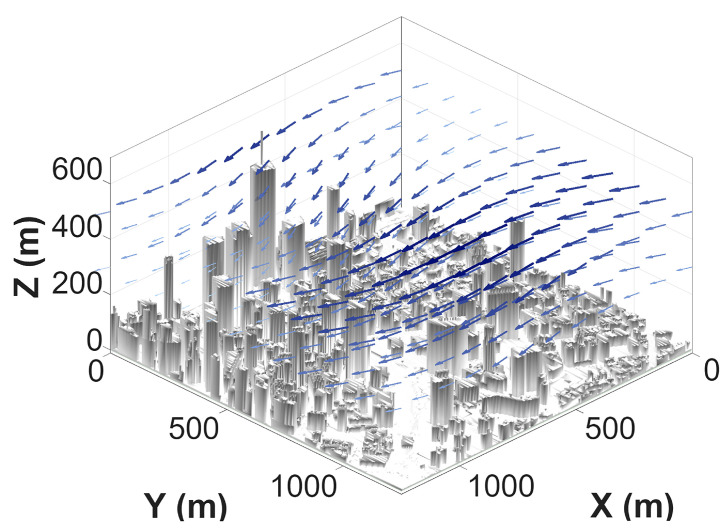
Three-dimensional wind field visualization coupled with complex urban building clusters. Arrows indicate wind direction, and arrow size indicates wind speed.

For clarity and to support the interpretation of simulation results, the spatial wind field is sampled on a grid and visualized using sparse wind vectors. Figure 1 and Figure 2 illustrate the 2D and 3D visualizations of the wind field, respectively. The arrow directions indicate wind orientation, while the color and length represent the magnitude at each grid point. Darker colors and longer vectors signify higher velocities, with the wind speed ranging from 5 m/s to 12 m/s, corresponding to the “Gentle Breeze” to “Strong Breeze” categories on the Beaufort scale. This specific range is supported by meteorological studies of low-altitude urban airspaces, where the “urban canyon effect” significantly amplifies ambient wind speeds between high-rise clusters [26]. According to aerospace engineering guidelines for small-to-medium UAVs, 12 m/s represents a critical operational threshold for maintaining control stability [27]. The selection of a steady-state model over a time-varying one is a deliberate methodological choice. As highlighted in recent MARL surveys [28], utilizing a time-averaged steady-state field is essential during the initial multi-agent coordination phase to isolate kinematic coupling effects and prevent high-frequency stochastic gusts from exacerbating environmental non-stationarity. While we utilize a steady-state 3D wind field as a foundational step to validate the kinematic-aware reward function, the logical progression of Figure 1, Figure 2 and Figure 3 explicitly addresses this setup: Figure 1 and Figure 2 establish the baseline isolated wind kinematics, while Figure 3 illustrates the coupled wind-terrain environment used for the final multi-agent training. However, we acknowledge that this represents a time-averaged simplification of complex urban turbulence.

Furthermore, a realistic urban architectural environment is constructed as the experimental scenario for multi-UAV collaborative path planning. This urban model is superimposed with the 3D wind field within a unified coordinate system to create a comprehensive simulation environment. Figure 3 provides a schematic of the urban 3D scene with the superimposed wind field, where gray entities represent buildings and blue vectors visualize the wind field distribution.

### 2.3. State Space and Action Space

In this study, RL is utilized to optimize action selection for multi-UAV path planning. To facilitate effective path planning and RL training within a discrete 3D grid environment, the agent’s action space is discretized into a 26-neighbor movement set. To accurately characterize the impact of wind field disturbances on UAV trajectories, the state transition model is formulated by superimposing the drift displacement caused by the wind field onto the intentional displacement generated by the 26-neighbor actions. Let the position of the UAV in the 3D grid space at time step *t* be denoted as:

(3) $$p_{t}=\left(x_{t},y_{t},z_{t}\right)$$

In this study, each UAV is abstracted as a point agent located at the center of a grid cell. The corresponding displacement vector for action $a_{t}$ is given by:

(4) $$\Delta p\left(a_{t}\right)=\left(\Delta x,\Delta y,\Delta z\right)$$

To account for the displacement induced by the wind field, the grid resolution *l* (measured in m/cell) is introduced as:

(5) l=gridresolution

The relationship between the physical coordinates and the grid indices is expressed as:

(6) $$r_{t}=p_{t}\cdot l$$

Consequently, the intentional displacement of the 26-neighbor actions within the physical coordinate system can be defined as:

(7) $$\Delta r_{action}=\Delta p\left(a_{t}\right)\cdot l$$

The 3D wind field is modeled as a velocity vector field:

(8) $$V_{wind}\left(r\right)={\left[U\left(r\right),V\left(r\right),W\left(r\right)\right]}^{T}$$

where r represents the physical coordinate position. Given a single time step duration Δt, the drift displacement caused by the wind field at the current position $r_{t}$ during step *t* is formulated as:

(9) $$\Delta r_{wind}=V_{wind}\left(r_{t}\right)\cdot \Delta t$$

Therefore, the state transition considering wind field disturbances can be represented as a 3D vector summation:

(10) $$r_{t+1}=r_{t}+\Delta r_{action}+\Delta r_{wind}$$

Subsequently, $r_{t+1}$ is mapped back to the 3D grid index to obtain the next state $p_{t+1}$:

(11) $$p_{t+1}=\mathrm{round}\left(\frac{r_{t+1}}{l}\right)$$

Assuming the system contains *M* UAVs, the individual state of the *i*-th UAV at time step *t* is denoted as $s_{t}^{i}$, and the joint state space of the environment is defined as:

(12) $$S_{t}=\left\{s_{t}^{1},s_{t}^{2},\ldots ,s_{t}^{M}\right\}$$

where $S_{t}$ denotes the collection of all UAV individual states maintained by the environment at time step *t*. It is used to update the multi-UAV environment and check inter-UAV interactions, whereas each agent receives only its own local state $s_{t}^{i}$ as the neural network input under the IQL formulation.

In practical sensor-driven path planning frameworks, the individual state $s_{t}^{i}$ serves as a comprehensive perceptual vector. It encapsulates the current kinematic data perceived by onboard sensors, including the relative distances to dynamic obstacles, the proximity to neighboring UAVs, and the localized wind velocity vectors $V_{wind}\left(r_{t}\right)$ detected by wind sensors. By formulating these multi-dimensional sensory inputs as the MDP state space, the DRL agent acts as an intelligent processing hub, directly mapping raw environmental perceptions to optimal collision-free maneuvers.

To make the observation space fully reproducible, the individual observation of the *i*-th UAV at time step *t* is explicitly defined as a 9-dimensional vector:

(13) $$s_{t}^{i}={\left[\Delta x_{g,t}^{i},\Delta y_{g,t}^{i},\Delta z_{g,t}^{i},d_{obs,t}^{i},d_{uav,t}^{i},U_{t}^{i},V_{t}^{i},W_{t}^{i},v_{wind,t}^{i}\right]}^{T}$$

The first three components represent the relative displacement from the current position of the *i*-th UAV to its assigned target in the global coordinate frame:

(14) $$\Delta x_{g,t}^{i}=x_{goal}^{i}-x_{t}^{i},\;\Delta y_{g,t}^{i}=y_{goal}^{i}-y_{t}^{i},\;\Delta z_{g,t}^{i}=z_{goal}^{i}-z_{t}^{i}$$

A comprehensive summary of the 9-dimensional observation vector is provided in Table 2.

All UAVs use the exact same observation definition, component ordering, and coordinate frames. The absolute UAV position is not directly used as a neural-network input feature; rather, it is implicitly utilized by the environment to compute the relative target vector, local wind components, and distances. Furthermore, heading and action histories are excluded to maintain a strictly memoryless Markov property. To ensure gradient stability, the raw observations are processed using clipping and Min-Max normalization. Specifically, the local distance features ($d_{obs,t}^{i}$ and $d_{uav,t}^{i}$) are truncated at the maximum sensing range of 15.0 m. Subsequently, all 9 components are normalized into a [−1,1] range based on their respective theoretical or physical boundaries before being fed into the neural network.

Accordingly, the individual action selected by the *i*-th UAV at time step *t* is denoted as $a_{t}^{i}$, while the joint action of the multi-UAV system is expressed as:

(15) $$A_{t}=\left\{a_{t}^{1},a_{t}^{2},\ldots ,a_{t}^{M}\right\}$$

where $A_{t}$ denotes the collection of individual actions executed simultaneously by all UAVs at time step *t*. The action collection $A_{t}$ is an environment-transition variable used to apply wind-induced motion and check collisions, rather than a centralized decision variable.

**Figure 4:**
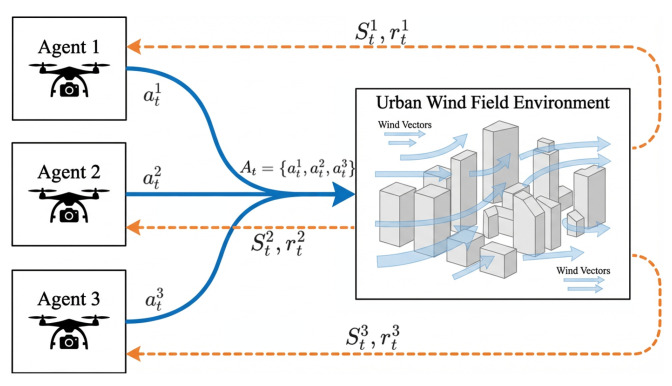
The proposed Agent–Environment interaction framework for multi-UAV cooperative path planning. Blue solid lines indicate the joint action vector $A_{t}$ executed in the urban wind field environment, while orange dashed lines represent the individual state and reward feedback ($S_{t}^{i},r_{t}^{i}$) returned to each specific DRL agent.

As shown in Figure 4, the proposed multi-UAV learning process follows a decentralized IQL formulation. Each UAV selects its action according to its own local observation:

(16) $$a_{t}^{i}∼\pi_{i}\left(\cdot |s_{t}^{i}\right)$$

where $\pi_{i}$ denotes the local policy of the *i*-th UAV. The environment receives the action collection and updates the UAV state collection according to:

(17) $$S_{t+1}∼P_{env}\left(S_{t+1}|S_{t},A_{t}\right)$$

where $P_{env}$ denotes the environment transition, including the 26-neighbor action displacement, wind-induced drift, obstacle checking, and inter-UAV collision checking. After the transition, each UAV receives its own local feedback:

(18) $$\left(s_{t+1}^{i},r_{t}^{i},d_{t}^{i}\right)$$

where $s_{t+1}^{i}$, $r_{t}^{i}$, and $d_{t}^{i}$ denote the next local observation, local reward, and termination flag of the *i*-th UAV, respectively.

The Q-network of the *i*-th UAV takes $s_{t}^{i}$ as input and estimates action values over its own discrete action set:

(19) $$Q_{i}\left(s_{t}^{i},a;\theta_{i}\right),\;a\in A_{i}$$

Thus, each UAV independently maintains and updates its own action-value function:

(20) $$Q_{i}\left(s_{t}^{i},a_{t}^{i};\theta_{i}\right)$$

**Figure 5:**
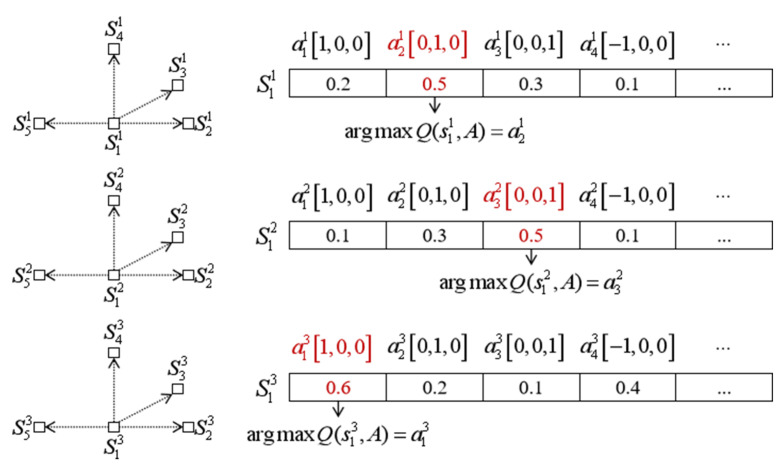
Mechanism of decentralized state representation and local action selection for individual agents.

As intuitively illustrated in Figure 5, this formulation ensures that the learning variables used for policy evaluation and action selection are consistently defined at the individual UAV level.

### 2.4. Reward Function

In the context of multi-UAV path planning, the design of the reward function is of paramount importance as it guides the agents toward learning optimal policies. To enhance the autonomous exploration capability of the agents and ensure a thorough investigation of the state space, an ϵ-greedy strategy is integrated into the learning process. This strategy balances exploration and exploitation by allowing each agent to select a random action with a probability of ϵ and the current optimal action with a probability of 1−ϵ. Specifically, when the action of the *i*-th agent is chosen stochastically, the selection probability is defined as ϵ/|A(s)|. Conversely, if $a_{t}^{i}$ corresponds to the optimal action, the probability is formulated as 1−ϵ+ϵ/|A(s)|, where 1−ϵ represents the probability of intentional exploitation and ϵ/|A(s)| accounts for the probability of selecting the optimal action through random exploration. If $a_{t}^{i}$ is neither the optimal action nor selected randomly, the policy $\pi (a_{t}^{i}|s_{t}^{i})=0$, indicating that such an action will not be executed. This mechanism is defined as follows:

(21) $$\pi \left(a_{t}^{i}|s_{t}^{i}\right)=\left\{\begin{array}{cc} 1-\epsilon +\frac{\epsilon }{\left|A(s)\right|}, & \mathrm{if}\;a_{t}^{i}={\text{arg max}}_{a}\;Q_{i}\left(s_{t}^{i},a\right)\\ \frac{\epsilon }{\left|A(s)\right|}, & \mathrm{otherwise} \end{array}\right.$$

where A(s) denotes the set of all feasible actions in state *s*, and |A(s)| represents the cardinality of this set (i.e., the number of available actions). The term $Q_{i}\left(s_{t}^{i},a\right)$ signifies the action-value function for the *i*-th agent taking action *a* in state $s_{t}^{i}$.

Regarding the reward structure, the formulation incorporates several critical factors: the number of steps taken by the agent, the alignment between the action direction and the wind field, the proximity to obstacles, potential collisions, and the attainment of the goal. To incentivize the agents to move toward their respective target points, a small positive reward is granted for reducing the distance to the goal, while a negative penalty is imposed for each step taken to encourage efficiency. Consequently, the reward increases as the agent approaches the target point and minimizes the total steps. This relationship can be mathematically expressed as:

(22) $$R_{dist}=\omega_{1}\left(d\left(p_{t-1}^{i},p_{goal}^{i}\right)-d\left(p_{t}^{i},p_{goal}^{i}\right)\right)-c_{step}$$

where $c_{step}=0.01$ is the time penalty per step designed to promote shorter decision sequences. This specific scalar was empirically selected to provide a gentle heuristic push toward optimal paths without overshadowing the primary task reward ($R_{goal}=100$). Preliminary sensitivity analyses indicate that the system’s convergence stability is robust to minor variations (e.g., within [0.005,0.05]). However, it is fundamentally sensitive to order-of-magnitude changes: an excessively large penalty (e.g., $c_{step}>1.0$) disrupts the gradient landscape, potentially inducing “reward hacking” where agents intentionally collide to terminate the episode prematurely and halt penalty accumulation. The term $d(p_{t}^{i},p_{goal}^{i})$ denotes the Euclidean distance from the current position to the target point for agent *i* in state $s_{t}^{i}$, and $\omega_{1}=0.1$ is a weighting factor used to balance the distance reward.

Considering the influence of wind field disturbances, agents moving in the direction of the wind can achieve higher speeds and improved energy efficiency. Therefore, the angle between the agent’s action direction and the wind flow is integrated into the reward function. A smaller angle θ results in a larger cosine value cosθ, yielding a higher reward, which is formulated as follows:

(23) $$R_{wind}=\omega_{2}\cos\theta \left(a_{t}^{i},V_{wind}\right)$$

where $\theta (a_{t}^{i},V_{wind})$ represents the angular difference between the intended direction of action for agent *i* at time step *t* and the local wind velocity vector. The associated weight $\omega_{2}$ is set to 0.5.

To facilitate effective obstacle avoidance, substantial negative rewards are assigned for collisions, while proximity-based penalties are applied to keep the agents at a safe distance from obstacles. The distance-dependent penalty for obstacles is expressed as:

(24) $$f\left(d\right)=\left\{\begin{array}{cc} 0, & d\geq d_{\mathrm{safe}}\\ \frac{1}{d+0.1}, & d<d_{\mathrm{safe}} \end{array}\right.$$

In this formulation, f(d)=0 when $d\geq d_{\mathrm{safe}}$, while f(d) increases significantly as the agent approaches an obstacle within the safety threshold $d<d_{\mathrm{safe}}$. To maintain strict dimensional consistency, the constant 0.1 in the denominator represents a physical distance of 0.1 m, which functions as a smoothing factor to prevent singularity (division by zero) when *d* approaches zero. This safety threshold $d_{\mathrm{safe}}$ implicitly accounts for the physical boundary and volumetric dimensions of the UAVs, ensuring collision-free maneuvers without treating the agent as a dimensionless point. The proximity penalty for structural obstacles increases as the agent moves closer, which can be mathematically expressed as:

(25) $$R_{obs}=-c_{obs}C_{obs,t}^{i}-\omega_{3}\sum_{j}f\left(d\left(p_{t}^{i},o_{j}\right)\right)$$

where $d(p_{t}^{i},o_{j})$ denotes the distance between agent *i* and the *j*-th obstacle. The coefficients $\omega_{3}$ and $c_{obs}$ are set to 0.02 and 20, respectively. Collision penalties are assigned at the individual UAV level. The obstacle collision indicator of the *i*-th UAV is defined as:

(26) $$C_{obs,t}^{i}\in \left\{0,1\right\}$$

When the *i*-th UAV collides with an obstacle or leaves the valid workspace, $C_{obs,t}^{i}=1$. Otherwise, $C_{obs,t}^{i}=0$.

For inter-UAV collisions, the collision indicator is defined as:

(27) $$C_{ij,t}=I\left(d_{ij,t}<d_{\mathrm{safe}}\right),\;i\neq j$$

where $d_{ij,t}=\left|\right|r_{t}^{i}-r_{t}^{j}{\left|\right|}_{2}$, $C_{ij,t}$ indicates whether the *i*-th UAV and the *j*-th UAV collide at time step *t*, $d_{ij,t}$ is their Euclidean distance, and $d_{\mathrm{safe}}$ is the safety-distance threshold. The inter-UAV collision indicator of the *i*-th UAV is then defined as:

(28) $$C_{uav,t}^{i}=I\left(\sum_{j\neq i}C_{ij,t}>0\right)$$

Therefore, if the *i*-th UAV and the *j*-th UAV collide, both UAVs receive the inter-UAV collision penalty in their own local rewards. UAVs not involved in that collision do not receive this penalty. If multiple collisions occur at the same time step, the penalty of each UAV is determined by its own collision indicators $C_{obs,t}^{i}$ and $C_{uav,t}^{i}$.

The inter-UAV reward component for agent *i* is thus:

(29) $$R_{uav}=-c_{uav}C_{uav,t}^{i}-\omega_{4}\sum_{j\neq i}f\left(d_{ij,t}\right)$$

where the weighting factors $\omega_{4}$ and $c_{uav}$ are assigned values of 0.03 and 15, respectively.

Upon reaching the target destination, agent *i* receives a significant positive reward $R_{goal}$. In all other instances, the composite reward is the summation of the aforementioned components, expressed as:

(30) $$r_{t}^{i}=\left\{\begin{array}{cc} R_{goal}, & \mathrm{if}\;\mathrm{target}\;\mathrm{is}\;\mathrm{reached}\\ R_{dist}+R_{wind}+R_{obs}+R_{uav}, & \mathrm{otherwise} \end{array}\right.$$

The reward $r_{t}^{i}$ is the local reward used for the Q-network update of the *i*-th UAV. Each UAV updates its action-value function using its own local reward rather than the summed reward of all UAVs. The summed reward of the multi-UAV system is recorded as:

(31) $$R_{t}^{sys}=\sum_{i=1}^{M}r_{t}^{i}$$

where $R_{t}^{sys}$ denotes the summed reward used to describe the overall training performance of the multi-UAV system. It is not used as the TD target for any individual Q-network. The learning update of the *i*-th UAV is based on $r_{t}^{i}$. This comprehensive reward scheme guides the multi-agent system to achieve efficient and collaborative path planning by accounting for diverse environmental and operational factors.

Given the varying scales and magnitudes of different reward components, normalization is necessary to prevent any single term from dominating the learning process. The normalized reward is defined as follows:

(32) $$R_{norm}=\frac{R-R_{min}}{R_{max}-R_{min}}$$

where $R_{max}$ and $R_{min}$ are the maximum and minimum cumulative returns observed during the training phase over multiple episodes. By normalizing the composite reward, the feedback signal becomes numerically comparable across different agents, thereby facilitating global learning stabilization and policy optimization for the multi-UAV system.

### 2.5. Improved NPD3QN Algorithm

In urban low-altitude environments characterized by wind field disturbances, cooperative path planning for multiple UAVs involves a high-dimensional joint state space. Under this setting, traditional tabular reinforcement learning struggles to generalize effectively in large-scale state spaces. Deep neural networks, however, can achieve high-dimensional mapping of action values through function approximation, offering an effective solution to this problem. This paper introduces deep neural networks to address low-altitude environments containing wind field disturbances, a method referred to as deep reinforcement learning.

The Deep Q-Network (DQN) is a representative algorithm in the field of deep reinforcement learning. Its core objective is to minimize the loss function, thereby reducing the discrepancy between the estimated value and the actual return. However, DQN relies on a single neural network to perform the Q-value estimation task, which leads to overestimation of Q-values and deviation from the true values. Double DQN (DDQN) innovatively adopts a dual-network architecture, decoupling action selection and value evaluation into two separate networks, which successfully resolves the Q-value overestimation issue. Dueling Double DQN (D3QN) builds upon the dual-network architecture of DDQN by further designing the network structure into two parallel branches. One branch focuses on evaluating the value of the state itself, while the other emphasizes measuring the relative advantage of taking different actions in a specific state. D3QN not only inherits the advantages of DDQN in reducing estimation bias and improving stability but also enables a more detailed and precise analysis of the value of various states and actions in complex urban low-altitude environments. This allows the agent to make wiser and more efficient decisions in continuous and high-dimensional spaces, greatly expanding the potential of deep reinforcement learning in practical applications.

Traditional D3QN algorithms only consider single-step updates, making it difficult to effectively utilize long-term return information. To enhance the ability to characterize long-term returns, this paper introduces an *N*-step update strategy based on the D3QN algorithm. At each decision point, the agent estimates the cumulative reward over multiple future steps and the state value estimate after the *N*-th step to update the value function of the current state. By comprehensively considering future information, the agent can plan an action path that is relatively optimal over a future period, avoiding the loss of larger subsequent returns due to immediate short-term interests. The return equation is expressed as:

(33) $$y_{t}=\sum_{k=0}^{N-1}\gamma^{k}r_{t+k}^{i}+\gamma^{N}\left(1-\mathrm{done}\right)Q_{target}\left(s_{t+N},\arg\;\max_{a}\;Q_{online}\left(s_{t+N},a;\theta_{i}\right);\theta_{i}^{-}\right)$$

where $r_{t}^{i}$ is the immediate reward obtained by agent *i* at time step *t*. γ is the discount factor, used to balance the influence of current and future rewards on decision-making. $s_{t+N}$ is the state of agent *i* after *N* steps from the current state. done is the termination flag, which takes the value of 1 when termination conditions such as reaching the target or collision occur, and 0 otherwise. $Q_{online}$ is the action value function output by the online Q-network of agent *i*, with parameters $\theta_{i}$. $Q_{target}$ is the action value function output by the target Q-network of agent *i*, with parameters $\theta_{i}^{-}$.

The PER mechanism is a strategy in reinforcement learning for optimizing experience sampling. Compared with uniform sampling, the PER mechanism uses the Temporal Difference-error (TD-error) as a metric to evaluate the priority of sampled data. The TD-error refers to the difference between the online Q-value and the target Q-value; a larger error indicates that the data is more helpful for updating network parameters. Therefore, the larger the absolute value of the TD-error of a sample, the greater its guiding role in learning, and the more likely it is to be sampled. On this basis, the PER mechanism can accelerate the convergence speed of the network. The priority setting based on TD-error can be expressed as:

(34) $$\delta =y_{t}-Q_{online}\left(s_{t},a_{t};\theta_{i}\right)$$

where δ represents the error of the current experience, that is, the deviation between the agent’s estimate of the optimal behavior in the current state and the actual return. To ensure that all experiences have a chance to be sampled and to avoid the priority being zero due to excessively small errors, a small offset ζ is added to the priority calculation of the *l*-th experience, expressed as:

(35) $$p_{l}=\left|\delta_{l}\right|+\zeta$$

The existence of priority directly affects the sampling probability of the *l*-th experience, namely:

(36) $$P\left(l\right)=\frac{p_{l}^{\alpha }}{\sum_{k}p_{k}^{\alpha }}$$

where α is a tuning parameter used to control the degree of influence of priority on sampling. When α=0, PER degenerates into uniform sampling; when α>0, experiences with larger TD-errors are assigned higher sampling probabilities. When adopting the PER mechanism, non-uniform sampling changes the probability distribution of samples being replayed. To mitigate the sampling bias caused by this, importance sampling weights are usually introduced to weight and correct the gradient update, expressed as:

(37) $$w_{l}=\frac{{\left(N\cdot P\left(l\right)\right)}^{-\beta }}{\max_{j}w_{j}}$$

where *N* is the size of the experience replay buffer. The hyperparameter β controls the strength of the importance sampling correction. To explicitly mitigate the variance amplification effects caused by extreme priority distributions during early learning, β is linearly annealed to 1.0 as training progresses. This ensures that the non-uniform sampling bias is fully compensated for in the later stages, effectively stabilizing the gradient updates.

However, when the priority distribution becomes highly skewed, extremely small replay probabilities may induce excessively strong importance sampling correction, which in turn enlarges the fluctuation of weighted updates and undermines training stability. To improve the numerical robustness of the replay mechanism, a stabilization treatment is further introduced in this study. The effective replay probability is regularized as:

(38) $$\tilde{P}\left(l\right)=\max\left(P\left(l\right),\epsilon_{p}\right)$$

where $\epsilon_{p}$ is a small probability floor used to prevent excessively small replay probabilities. Based on $\tilde{P}\left(l\right)$, the stabilized importance sampling weight is computed as:

(39) $${\hat{\omega }}_{l}={\left(N\cdot \tilde{P}\left(l\right)\right)}^{-\beta }$$

and the final normalized weight is defined as:

(40) $$\omega_{l}=\frac{\mathrm{clip}({\hat{\omega }}_{l},\omega_{min},\omega_{max})}{\max_{j}\mathrm{clip}\left({\hat{\omega }}_{j},\omega_{min},\omega_{max}\right)}$$

where $\omega_{min}$ and $\omega_{max}$ denote the lower and upper clipping bounds of the importance sampling weights, respectively. By constraining the correction magnitude associated with extremely small replay probabilities, this treatment alleviates the variance amplification effect under severely imbalanced replay distributions and improves the stability of weighted updates.

In multi-UAV scenarios with wind field disturbances and building constraints, the stochastic interaction between the agent and the environment leads to an imbalance in the proportion of positive and negative experiences in the replay buffer. This imbalance directly affects the learning direction of the agent, making it prone to repeatedly sampling a large number of negative experiences, thereby reducing the reuse efficiency of key experiences. This paper improves the PER mechanism by introducing a combined mechanism of marking experience types, controlling the probability of entering the replay buffer, and weighting sampling weights.

This paper denotes a single experience transition as $e_{t}$ and defines a positive experience indicator variable $I_{pos}$. To provide a precise and granular evaluation of environmental feedback, experience transitions are systematically categorized into four classes: (1) *Task-progression transitions* (reaching the target or safely reducing the distance to the goal); (2) *Near-miss transitions* (avoiding collision but entering the critical safety margin $0<d<d_{\mathrm{safe}}$); (3) *Neutral/Inefficient-but-safe transitions* (collision-free but failing to progress toward the goal); (4) *Terminal collision transitions* (crashing into obstacles or peers).

To resolve any logical ambiguity regarding safety-critical learning, $I_{pos}$ is assigned a value of 1 for both task-progression and near-miss transitions. Because a near-miss does not result in a terminal failure, it enters the replay buffer with a probability of 1.0. Importantly, near-miss transitions incur a severe distance-dependent proximity penalty (as defined in Equation (24)). This massive penalty typically deviates significantly from the network’s current value estimation, generating an exceptionally large Temporal Difference (TD) error (δ). According to our mechanism (Equation (43)), the sampling probability of a near-miss sample is thus amplified twice: first by its intrinsically large TD-error base priority ($p_{l}$), and second by the positive weighting coefficient (η). Consequently, near-miss experiences organically dominate the sampling distribution, forcing the policy to rigorously learn the boundaries of safe clearance. Conversely, “negative” or “low-value” experiences ($I_{pos}=0$) are strictly defined as the combination of terminal collisions and neutral/inefficient transitions. While initially informative, an over-accumulation of terminal collisions causes gradient dominance by penalty outliers, and excessive neutral transitions dilute the density of goal-oriented signals, both leading to conservative policy freezing. Thus, these specific non-constructive experiences are intentionally down-sampled by the entry probability factor ρ. The current proportion of positive experiences $R_{pos}$ is defined as:

(41) $$R_{pos}=\frac{\sum_{k=1}^{N}I_{pos}^{k}}{N}$$

To avoid negative experiences accumulating excessively in the replay buffer and occupying capacity, this paper sets a lower probability for negative experiences to enter the buffer:

(42) $$P_{enter}\left(e_{t}\right)=\left\{\begin{array}{cc} 1, & \mathrm{if}\;I_{pos}=1\\ \rho , & \mathrm{if}\;I_{pos}=0 \end{array}\right.$$

where ρ is the probability of negative experiences entering the replay buffer. During sampling, this paper applies a positive weight to positive experiences on top of the base priority of the PER mechanism. First, the sample priority $p_{l}$ is defined according to the TD-error. On this basis, a positive experience weighting coefficient η is introduced, and the sampling probability is defined as:

(43) $$p_{l}^{*}=\left\{\begin{array}{cc} \eta \cdot p_{l}, & \mathrm{if}\;I_{pos}=1\\ p_{l}, & \mathrm{if}\;I_{pos}=0 \end{array}\right.$$

When $I_{pos}=1$, the sample priority is amplified η times, thereby increasing the sampling probability of positive experiences; when $I_{pos}=0$, the standard PER mechanism is used for sampling. By suppressing negative experiences entering the replay buffer and positively weighting positive experiences during the sampling phase, the proportion of positive experiences in the replay buffer gradually approaches the ideal ratio, improving sample utilization efficiency and reward convergence effects.

The D3QN algorithm integrating the *N*-step update strategy and the improved PER mechanism demonstrates a superior performance in multiple aspects. First, by introducing cumulative returns over multiple future time steps, the agent’s ability to perceive long-term returns is enhanced, effectively avoiding the local optimum problem caused by short-term reward driving in traditional single-step updates. Second, the improved PER mechanism suppresses the probability of negative experiences entering the replay buffer, reducing the occupation of the buffer by a large number of low-value samples, so that the proportion of positive experiences in the buffer is increased and closer to the preset ideal ratio. Moreover, positive experiences are positively weighted during the sampling phase, allowing them to obtain a higher sampling probability while maintaining the TD-error priority principle, thereby significantly improving the reuse frequency of key experiences and gradient update efficiency. This mechanism can effectively reduce invalid updates caused by repeated sampling of negative experiences, improving the stability and convergence speed of the training process.

Furthermore, the *N*-step update strategy and the improved experience replay mechanism are complementary. The former enhances the long-term return of value learning through return estimation over a longer time scale, while the latter stabilizes the training process by increasing the proportion and sampling frequency of high-value samples. When combined, the agent cannot only capture effective strategies leading to the target more quickly but also maintain stronger robustness and generalization capabilities under complex wind field disturbances and obstacle constraints.

In summary, the D3QN algorithm integrating the *N*-step update strategy and the improved PER mechanism (NPD3QN) exhibits improved performance in the simulated environment in terms of learning efficiency, convergence stability, and final planning quality, providing a more reliable decision-making method for path planning tasks of multiple UAVs in urban low-altitude environments with wind fields.

*Theoretical Justification for Task-Oriented PER:* In safety-critical urban navigation, an over-abundance of negative experiences (collisions) during early training stages can lead to “conservative policy freezing,” where agents fail to explore viable paths. The proposed NPD3QN regulates the sampling density of these experiences to prevent gradient dominance by penalty outliers. This effectively balances exploration efficiency and safety constraints, ensuring the collision rate converges to near-zero (as demonstrated in Section 3.5 and Section 3.6).

**Figure 6:**
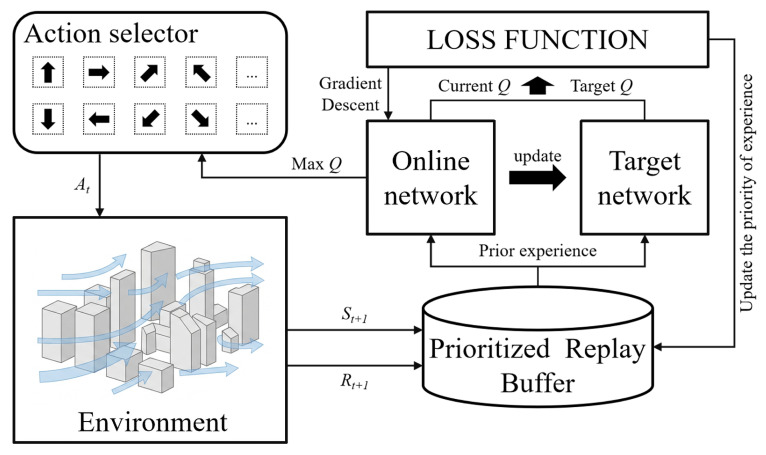
Algorithm framework of the proposed NPD3QN.

This paper applies the proposed method to perform path planning for agents, and the algorithm framework is shown in Figure 6.

### 2.6. Implementation Details and Hyperparameters

To ensure full reproducibility, the detailed configurations are provided. The multi-UAV system is trained using the Independent Q-Learning (IQL) paradigm. Each agent’s neural network processes a 9-dimensional state vector. To stabilize training, this state vector is scaled using Min-Max feature normalization into a [−1,1] range. The sensory range for obstacle and inter-UAV detection is assumed to be 15.0 m. The network consists of two shared hidden layers with 128 and 64 neurons respectively (ReLU activation), followed by a Dueling head separating state value and advantage streams, both with branch widths set to 32.

The training parameters are standardized across all runs: the Adam optimizer is employed with a learning rate of $1\times 10^{-4}$, a batch size of 64, a discount factor of γ=0.99, and a replay buffer capacity of $1\times 10^{5}$. The target network is updated every 500 steps. The ϵ-greedy exploration rate linearly decays from an initial value of 1.0 to a minimum of 0.05 over the first 20% of total training steps.

Regarding the kinematic environment, the key task parameters are explicitly defined: the grid resolution is l=1.0 m/cell, the discrete time step is Δt=0.1 s, the safety distance threshold is $d_{\mathrm{safe}}=1.5$ m, and the terminal reward is $R_{goal}=100$. An episode reaches its termination condition when a UAV reaches its target, collides with an obstacle/another UAV, or exceeds the maximum episode length of 500 steps. All evaluation metrics are averaged over 5 independent random seeds.

To ensure a fair and rigorous comparison, the Multi-Agent Proximal Policy Optimization (MAPPO) baseline was implemented following the centralized training and decentralized execution (CTDE) architecture. Both the actor and critic networks share a similar backbone to NPD3QN, consisting of two hidden layers (128 and 64 neurons, ReLU activation). The actor utilizes a softmax output layer to handle the discrete 26-dimensional action space, while the centralized critic processes the global state (concatenating all agents’ local observations) during training to estimate the baseline value. Key hyperparameters for MAPPO were fine-tuned as follows: the Adam optimizer was employed with a learning rate of $5\times 10^{-4}$, the PPO clipping coefficient was 0.2, the entropy coefficient was 0.01, and the value-loss coefficient was 0.5. The PPO update cycle utilized a rollout length of 4000 steps, a minibatch size of 256, and 10 optimization epochs per update. The discount factor was γ=0.99, and Generalized Advantage Estimation (GAE) was applied with λ=0.95 to reduce variance. Finally, it is explicitly clarified that MAPPO was evaluated using the exact same reward structure, 3D wind-field configurations, 9-dimensional observation inputs, and total training budget across 5 independent random seeds to ensure strict benchmarking fairness.

## 3. Experiments

### 3.1. Parameter Sensitivity Experiments

In the proposed NPD3QN algorithm, the update steps *N*, the probability of negative experiences entering the replay buffer (ρ), and the sampling weight coefficient for positive experiences (η) exert significant influence on convergence speed, training stability, and final policy performance. The update steps *N* determine the quantity of future rewards incorporated into the update target. Specifically, a value that is too small leads to excessive reliance on short-term rewards and fails to fully utilize long-term reward information. Conversely, an excessively large *N* introduces significant cumulative estimation errors, thereby compromising the stability and performance of the algorithm. The probability ρ is employed to regulate the entry of negative experiences into the replay buffer. A smaller ρ reduces the influx of negative experiences, thereby increasing the relative proportion of positive experiences in the buffer. This mechanism enhances sample utilization efficiency and the density of effective updates during the initial training phase. However, an excessively low ρ results in insufficient sample diversity, which degrades the generalization capability of the policy in dynamic environments and renders performance more sensitive to environmental perturbations. Furthermore, the sampling weight coefficient η is utilized to increase the replay probability of positive experiences during the sampling phase. If η is too small, the sampling process fails to focus effectively on critical decision-making samples; if η is too large, the process becomes highly dependent on positive experience samples, leading to a loss of diversity and the emergence of overfitting.

To evaluate the impact of these key hyperparameters on the learning process and policy performance, and to establish a unified and reproducible configuration for subsequent comparative experiments, parameter sensitivity experiments were conducted to determine the optimal values for *N*, ρ, and η. To ensure a fair comparison, all experiments were performed under identical environmental conditions and training configurations, varying only the combinations of *N*, ρ, and η. The evaluation process was executed in three stages: first, the performance of various configurations was assessed over 200 training episodes to select the top 12 candidates; second, these 12 configurations were evaluated over 600 episodes to identify the top 4; finally, the 4 leading configurations were tested over 2000 episodes. The configuration exhibiting the highest performance ranking was selected as the final hyperparameter set for the algorithm.

Table 3 demonstrates the path planning performance under different parameter configurations across various training durations. As the number of training episodes increases, the success rate and average reward for each configuration exhibit an upward trend. However, distinct performance gaps persist between different combinations, indicating that these key hyperparameters significantly affect final policy performance and stability.

At 600 training episodes, while certain combinations achieve relatively high average rewards, their success rates remain suboptimal. This suggests that reward enhancement is not strictly equivalent to task completion capability, necessitating a joint evaluation of success rates and reward volatility. At 2000 episodes, the number of candidate configurations converges to a few optimal sets, with clearer stratification observed in terms of success rate, average reward, and reward fluctuation, providing a robust basis for final parameter determination.

**Figure 7:**
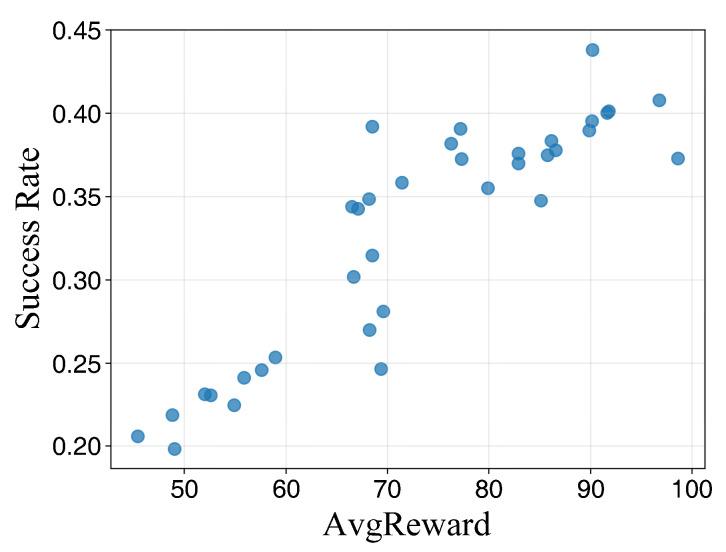
Scatter of performance for candidate parameter configurations.

**Figure 8:**
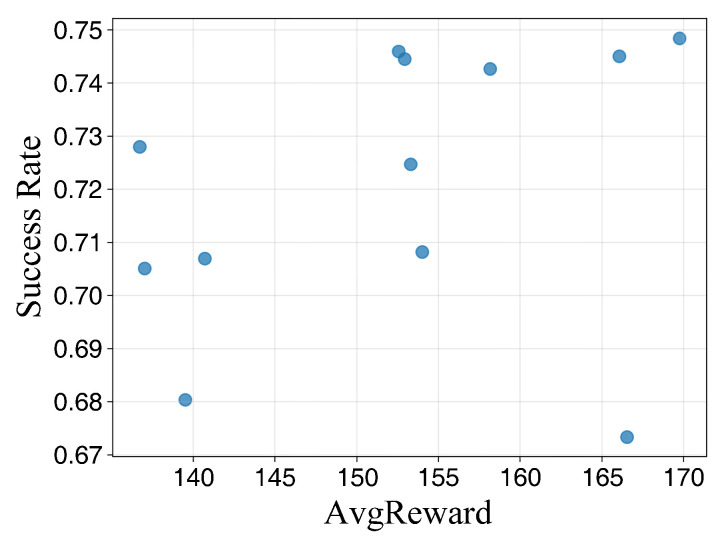
Scatter of performance for retained parameter configurations (top 12 evaluated).

**Figure 9:**
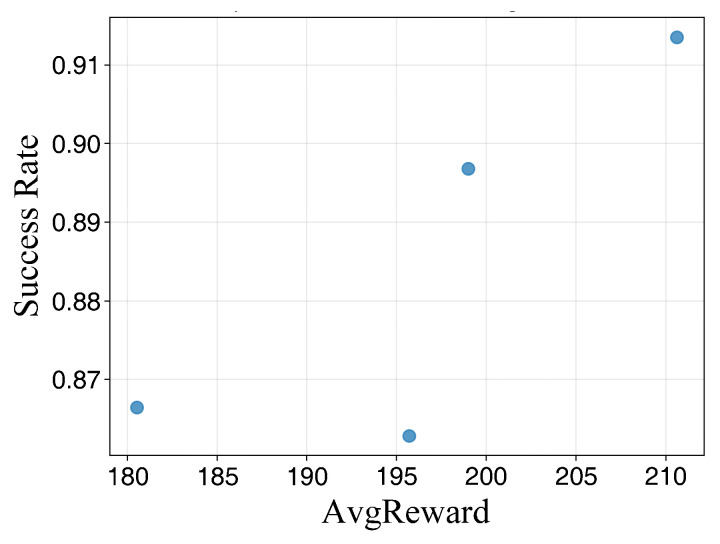
Scatter of performance for the final retained parameter configurations (final 4 evaluated).

The conclusions are further validated by the results presented in Figure 7, Figure 8 and Figure 9. At lower training episode counts, the data point distribution is relatively discrete but exhibits a general positive correlation, indicating that rewards effectively reflect task completion quality despite significant fluctuations. As training progresses, the point sets gradually cluster toward regions of high success rates and high rewards. Notably, isolated points with high rewards but low success rates are observed, corroborating the observation from Table 3 that single-metric evaluations may lead to misjudgment. Thus, parameter selection must simultaneously consider both success rates and reward levels. Under long-duration training, the scatter distribution becomes more concentrated, and the configurations located in the upper-right region correspond to higher reward values and success rates, indicating improved planning performance and reachability stability in the simulated environment.

**Figure 10:**
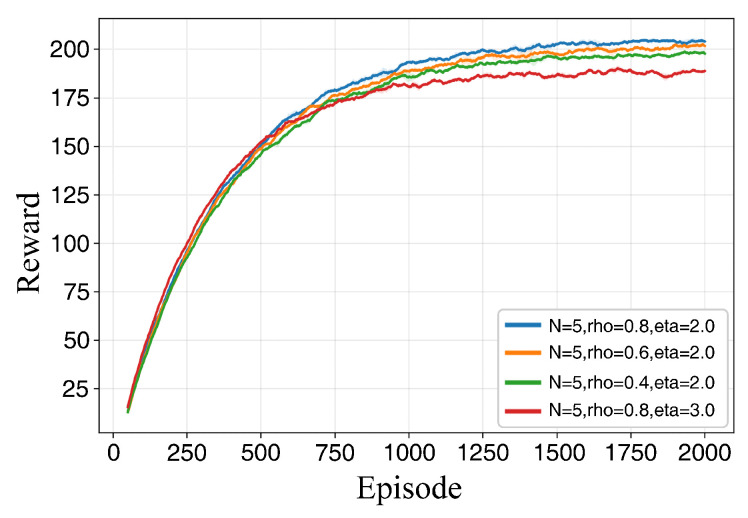
Reward convergence for the final retained parameter configurations.

**Figure 11:**
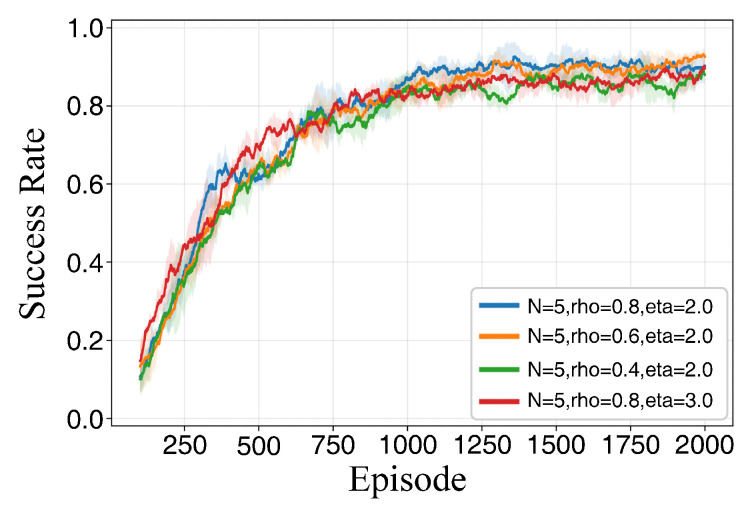
Success rate for the final retained parameter configurations.

Figure 10 and Figure 11 further distinguish the configurations from the perspective of training dynamics. As shown in Figure 10, all four candidate configurations follow a learning pattern characterized by a rapid initial increase followed by gradual stabilization. However, variations exist in reward convergence and the magnitude of late-stage oscillations. The selected configuration enters the stable phase more rapidly and maintains a higher converged reward with minimal oscillation. When ρ is excessively low, the final converged reward is lower, reflecting the constraints imposed by insufficient experience diversity on long-term performance. When η is excessively large, although the initial growth rate is competitive, the final reward convergence is lower and exhibits more pronounced fluctuations, suggesting that excessive sampling bias weakens the error-correction capability and hinders stable convergence. Correspondingly, Figure 11 illustrates that the success rates for all configurations increase and converge as training progresses. The superior configuration maintains a more stable and higher success rate in the mid-to-late stages, whereas configurations with low ρ or high η are more prone to periodic fluctuations and a declining trend in final stability levels.

Based on the aforementioned experimental results, the hyperparameters for the proposed NPD3QN algorithm are determined as N=5, ρ=0.8, and η=2.0. This configuration achieves stable comprehensive performance over long training durations and exhibits consistent advantages in terms of convergence speed, stability, and final performance. Consequently, it is adopted as the default hyperparameter setting for all subsequent comparative experiments in this study.

### 3.2. Importance-Sampling Stability Analysis

**Figure 12:**
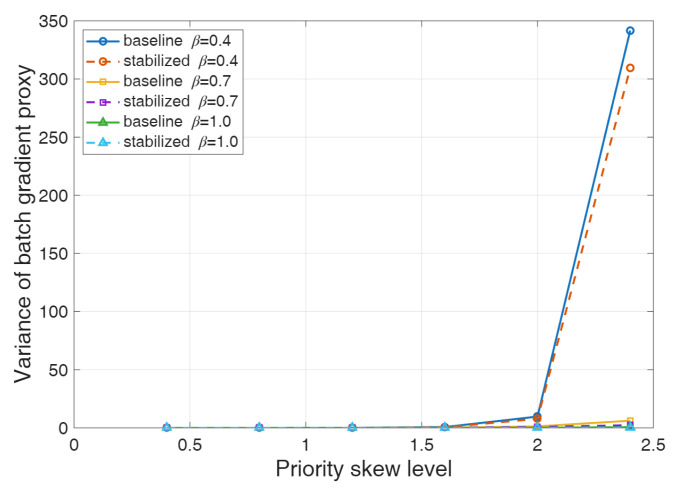
Variance of weighted mini-batch updates under different priority skew levels.

**Figure 13:**
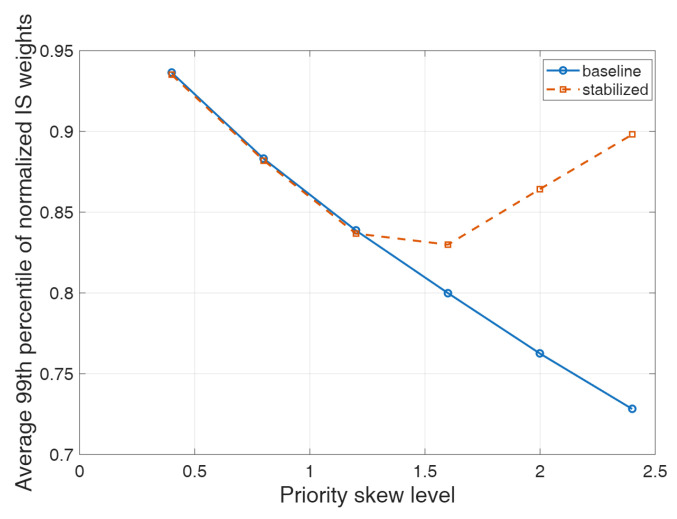
Sampling mass of the top 1% high-priority samples under different priority skew levels.

To further investigate the stability of the importance-sampling correction in prioritized experience replay under extreme priority distributions, an additional importance-sampling stability analysis was performed, as illustrated in Figure 12. Specifically, priority distributions with different skew levels were constructed to examine how the fluctuation of weighted mini-batch updates evolves as the sampling probability becomes increasingly concentrated on a small subset of high-priority samples. In this analysis, the variance of weighted mini-batch updates was adopted as the primary evaluation metric to quantify the effect of importance-sampling correction on the stability of parameter updates. Meanwhile, as shown in Figure 13, the sampling mass of the top 1% high-priority samples is used to characterize the concentration degree of the replay distribution.

The results indicate that, as the skewness of the priority distribution increases, the variance of weighted mini-batch updates under the original importance-sampling correction rises markedly, revealing a pronounced variance amplification effect under extreme priority distributions. This tendency is particularly evident under medium and strong importance-sampling correction strengths. When the correction strength is set at a medium level, the variance of weighted mini-batch updates increases from 0.0028 under the low-skew condition to 6.1674 under the high-skew condition. When the correction strength is further increased, the corresponding value rises from 0.0035 to 0.6345. These findings indicate that, as the replay probability becomes increasingly concentrated on a limited number of high-priority samples, the original importance-sampling weights lead to rapidly intensified update fluctuations, thereby undermining the stability of the training process.

By contrast, after introducing the stabilization treatment, the variance of weighted mini-batch updates is substantially suppressed under high-skew conditions. When the skew level becomes relatively high, variance reduction can be observed under all correction strengths, with the most significant improvements achieved under medium and strong correction settings. Under the highest-skew condition, the variance of weighted mini-batch updates is reduced by approximately 62.5% and 65.0% under the medium and strong correction strengths, respectively. These results demonstrate that, when the replay distribution becomes highly imbalanced, the introduced stabilization mechanism can effectively alleviate the amplification of update fluctuations caused by importance-sampling weights, thus enhancing the robustness of the replay correction process.

Furthermore, the sampling mass of the top 1% high-priority samples increases continuously from approximately 2.2% under the low-skew condition to about 18–19% under the high-skew condition, indicating that the sampling probability becomes highly concentrated on a very small subset of samples. This observation confirms that the constructed priority distributions effectively capture the sampling characteristics of prioritized replay under extreme imbalance conditions. Under such circumstances, the original importance-sampling correction is more likely to induce a rapid increase in update variance, whereas the stabilized treatment can effectively suppress this trend. It is also worth noting that, under low-skew conditions, the difference between the original method and the stabilized method remains relatively small. This suggests that the proposed stabilization strategy primarily takes effect in severely imbalanced replay scenarios and does not significantly alter the basic update behavior of the algorithm under normal sampling conditions. Overall, the above results confirm that extreme priority distributions can amplify the fluctuation introduced by the importance-sampling correction, while the stabilization treatment adopted in this study effectively improves the numerical stability of the training process.

### 3.3. Algorithm Effectiveness Experiment

To evaluate the path planning performance and learning convergence characteristics of the proposed method, comparative experiments are first conducted in a wind-free environment. For a fair comparison, the environmental map, obstacle distribution, and hyperparameter settings are maintained consistently across all simulations. The start and target positions for each agent are fixed. The proposed NPD3QN algorithm is compared against four baseline methods: the traditional A* search algorithm, the standard DQN, the Dueling Double Deep Q-Network (D3QN), and Multi-Agent Proximal Policy Optimization (MAPPO). MAPPO is included as a widely adopted modern MARL baseline to rigorously evaluate multi-agent cooperative efficiency.

**Figure 14:**
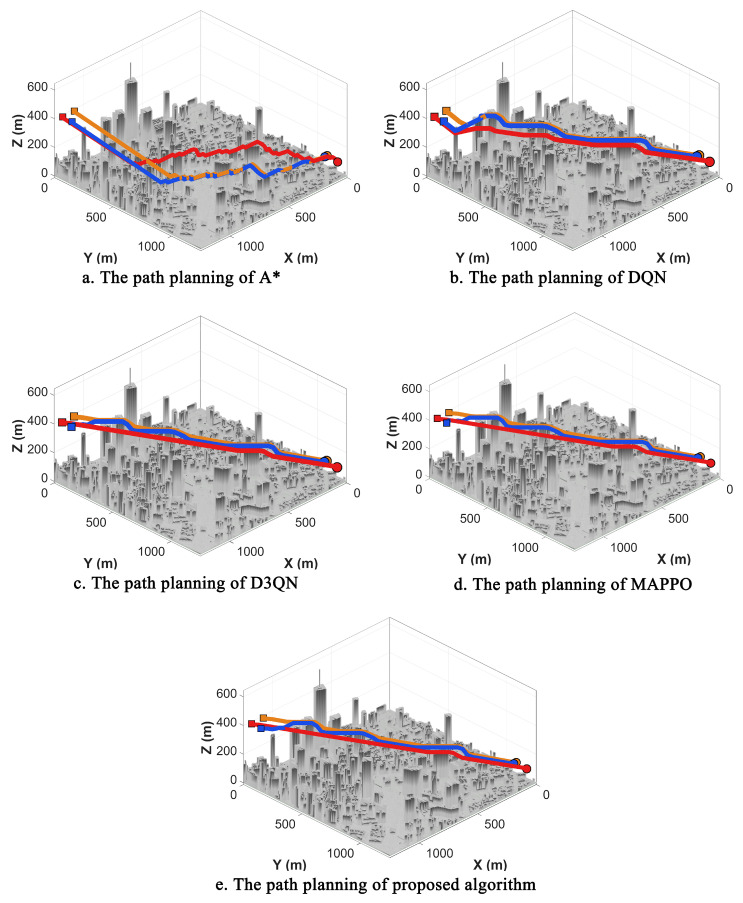
Path planning of A*, DQN, D3QN, MAPPO, and the proposed algorithm (no wind field).

**Figure 15:**
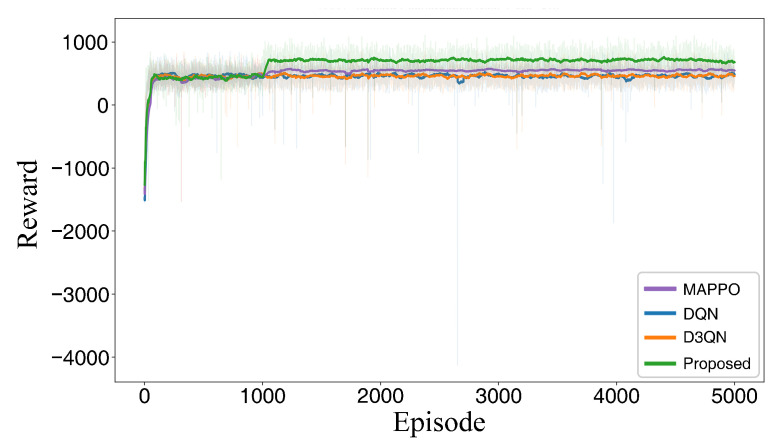
Reward convergence of MAPPO, DQN, D3QN, and the proposed algorithm (no wind field).

To ensure statistical rigor, all DRL models were trained and evaluated across 5 independent random seeds. Comprehensive analysis is performed based on the visual path planning results, path length metrics, Success Rate (SR), Collision Rate (CR), average computation time per step, and the reward convergence curves observed during training. Quantitative metrics are reported as Mean ± Standard Deviation to account for stochastic variances. The comparative path planning trajectories and reward convergence trajectories are illustrated in Figure 14 and Figure 15, respectively, while the quantitative statistical results for path length are summarized in Table 4.

The results demonstrate significant performance variations among the evaluated algorithms in the wind-free scenario. While the A* algorithm is capable of generating feasible paths, its search space expands exponentially when integrated with multi-UAV cooperative constraints, such as maintaining safety distances in complex obstacle environments. This phenomenon often leads to excessive turns and detours, resulting in a significantly larger total path length. In contrast, the reinforcement learning (RL)-based methods develop more holistic decision-making strategies through continuous environmental interaction. Under identical constraints, the RL-based approaches generally outperform traditional search methods by planning shorter, more efficient paths. A detailed comparison between the RL variants reveals that standard DQN exhibits relatively weak planning capabilities in multi-UAV cooperative scenarios, often producing redundant path segments. Although D3QN improves path quality and mitigates the overestimation issue—common in traditional Q-learning—through its enhanced value estimation mechanism, the proposed NPD3QN yields highly competitive and marginally shorter paths compared to the modern MAPPO baseline. To rigorously evaluate this performance margin, an independent two-sample *t*-test was conducted on the total path lengths across the 5 random seeds. The test yielded a *p*-value of 0.52 (p>0.05), indicating that the path-length advantage over MAPPO in the purely deterministic, wind-free setting is not statistically significant. Both NPD3QN and MAPPO demonstrate excellent, comparable spatiotemporal coordination in the absence of environmental disturbances. NPD3QN effectively coordinates action selection and collision avoidance among multiple agents, minimizing unnecessary detours and repetitive adjustments. Consequently, NPD3QN achieves the minimum total path length, showing its robust ability to leverage state information and cooperative constraints to maximize path planning efficiency in deterministic environments.

Regarding the training dynamics, the reward convergence curves highlight the superior stability and robustness of the NPD3QN algorithm. As shown in Figure 15, the proposed method demonstrates a more rapid reward increase during the early training stages and maintains a smoother convergence process, ultimately reaching a higher steady-state reward level. Conversely, both DQN and D3QN exhibit pronounced fluctuations during training and settle at lower asymptotic reward values. In summary, the NPD3QN algorithm not only outperforms the baseline methods in terms of planning efficiency but also demonstrates more reliable convergence trends. This robustness establishes a solid methodological foundation for subsequent applications in stochastic wind-field environments.

### 3.4. Wind Field Experiments

**Figure 16:**
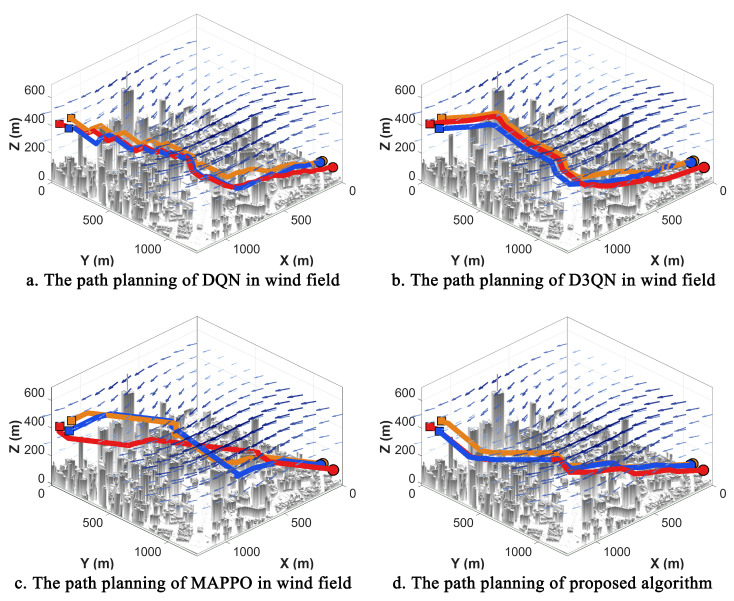
Path planning of DQN, D3QN, MAPPO, and the proposed algorithm (with wind field).

**Figure 17:**
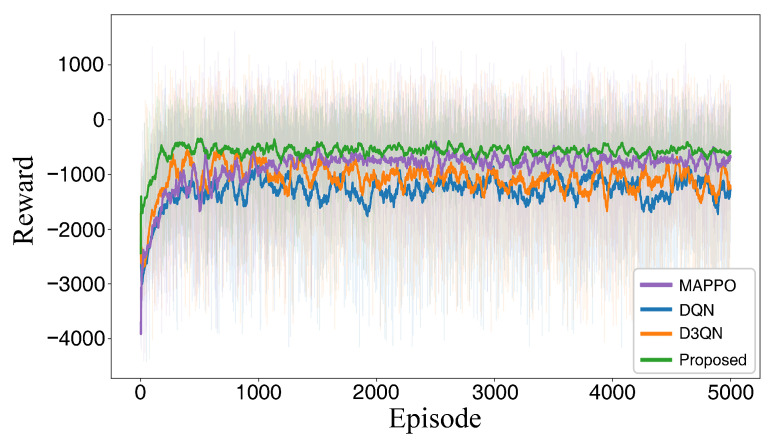
Reward convergence of MAPPO, DQN, D3QN, and the proposed algorithm (with wind field).

In actual urban environments, wind disturbances frequently induce UAV trajectory drift. This phenomenon causes paths that were optimal in windless conditions to suffer from significant yaw, increased detours, and even collision risks during execution, thereby imposing more stringent requirements on the robustness and generalization capabilities of multi-UAV cooperative planning strategies. To evaluate the performance of NPD3QN under such perturbations, comparative experiments were conducted in a simulated wind field to assess planning quality and training stability. The trajectory planning results and reward convergence profiles for DQN, D3QN, MAPPO, and NPD3QN are illustrated in Figure 16 and Figure 17, respectively, while the quantitative path length data are summarized in Table 5.

The statistical results of the path lengths in the wind field reveal a distinct performance hierarchy among the four reinforcement learning algorithms. At both the total cooperative path length and individual UAV path length levels, DQN consistently exhibits the largest planning lengths, followed by D3QN, while the proposed NPD3QN achieves the highest planning efficiency. Notably, this advantage remains consistent across all three UAVs. A cross-comparison with results from windless experiments indicates that all algorithms experience varying degrees of path length increments due to wind interference, confirming that environmental disturbances indeed escalate planning and execution complexity. However, compared to the baseline methods, NPD3QN maintains a significantly lower increment in path length. Specifically, NPD3QN reduces the total path length by approximately 19.9% compared to DQN and 11.7% compared to D3QN, demonstrating robust anti-disturbance capability and higher cooperative efficiency. The results in Table 5, averaged over 5 independent seeds, provide a more robust statistical comparison. While the modern MARL baseline, MAPPO, achieves a notable reduction in path length compared to DQN and D3QN, NPD3QN consistently maintains the most efficient trajectories with the smallest standard deviation. Specifically, NPD3QN exhibits a 6.7% improvement in total path length over MAPPO. To validate the statistical robustness of this advantage under environmental disturbances, an independent two-sample *t*-test was performed. The results confirmed that the path-length reduction achieved by NPD3QN over MAPPO is highly statistically significant (t=10.28,p<0.001). This rigorous statistical evidence firmly substantiates that our task-oriented experience replay mechanism more effectively captures optimal navigation strategies in wind-disturbed urban airspaces.

The visual planning results in Figure 16 further highlight the morphological disparities between the paths generated by these algorithms under identical wind conditions. Trajectories produced by DQN exhibit pronounced local oscillations and redundant steering maneuvers, resulting in a more tortuous overall path. This suggests that DQN’s action selection is highly susceptible to short-term reward fluctuations caused by wind perturbations, leading to increased path redundancy. While D3QN improves path smoothness and global consistency by mitigating some detours—attributable to its refined value estimation mechanism—NPD3QN consistently generates the most streamlined trajectories. By embedding wind field kinematics directly into the decision-making framework, NPD3QN learns to effectively exploit tailwinds while avoiding path increases caused by crosswinds. Consequently, it exhibits fewer abrupt turns and maintains higher directional alignment during obstacle avoidance and coordination, demonstrating a robust ability to preserve a stable global planning trend despite environmental noise.

The reward convergence curves further elucidate these performance disparities from a training perspective. Although all three algorithms experience rapid reward growth in the early stages, the DQN curve suffers from high-frequency fluctuations and a lower final stability level, indicating its struggle to formulate a stable policy under high-variance returns. While D3QN shows improvements in convergence level and stability, it still exhibits persistent oscillations. In contrast, the reward for NPD3QN ascends more rapidly, reaches the stability plateau significantly earlier, and maintains a higher reward level with minimal oscillation. These results confirm that NPD3QN can effectively learn robust strategies against disturbances and converge toward an optimal solution in a more stable manner.

### 3.5. Ablation Studies

**Figure 18:**
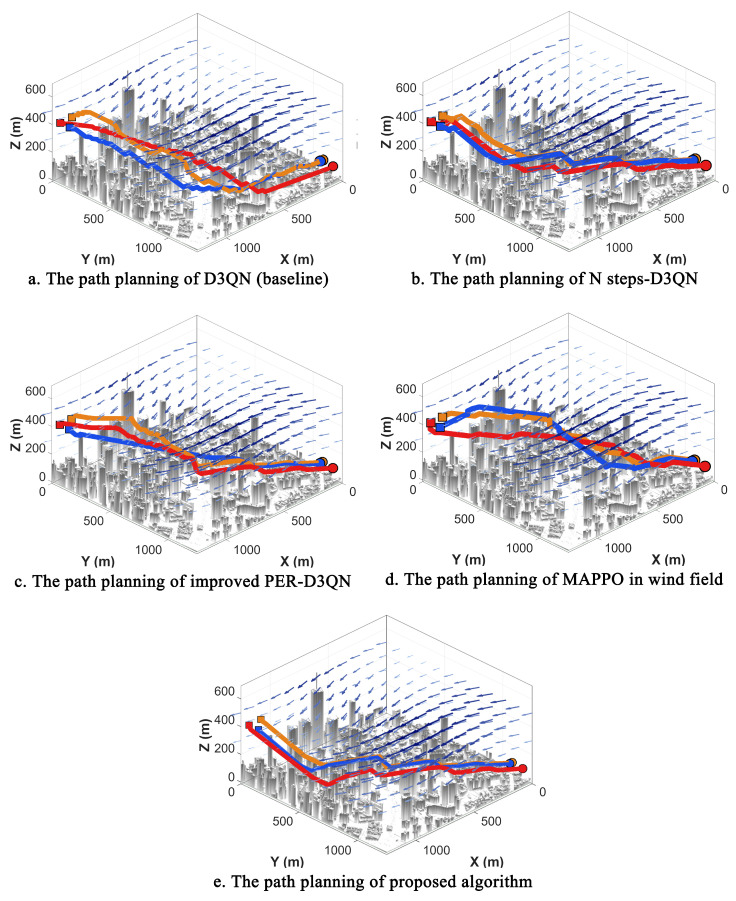
Path planning of D3QN, N steps-D3QN, improved PER-D3QN, MAPPO, and the proposed algorithm (with wind field).

**Figure 19:**
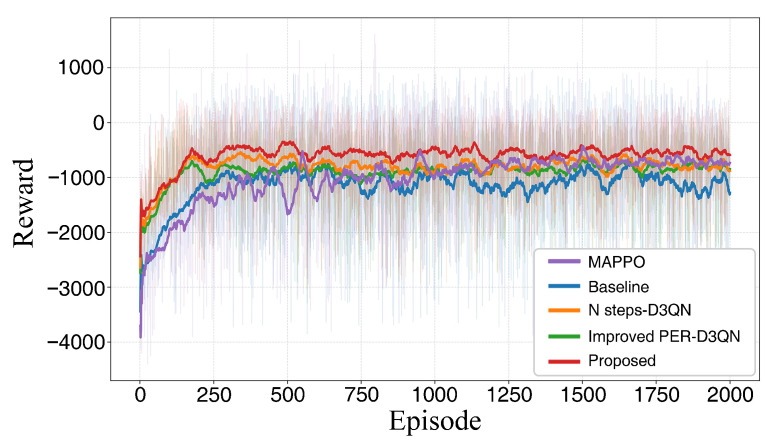
Reward convergence of MAPPO, D3QN, N steps-D3QN, improved PER-D3QN, and the proposed algorithm (with wind field).

Since NPD3QN integrates several enhancement mechanisms onto the D3QN framework, the overall performance gains may stem from the independent contributions of individual modules or their synergistic effects. To isolate the sources of improvement and avoid ambiguity, ablation studies were conducted to verify each module systematically. Using D3QN as the baseline, we constructed comparison groups by independently introducing the *N*-step update strategy, the improved PER mechanism, and the full integration of both (NPD3QN). All experiments were repeated under identical environmental configurations and task constraints. The planning results and reward convergence curves for these combinations are presented in Figure 18 and Figure 19, with the corresponding path length data provided in Table 6.

As illustrated in the expanded Table 6, the synergistic effect of the N-step update and improved PER mechanism is evident across multiple dimensions. While the N-step strategy primarily enhances path efficiency, the improved PER mechanism is a critical driver for mission safety. Specifically, the Collision Rate (CR) drops significantly from 12.5% (Baseline) to 3.6% (NPD3QN), while the Success Rate (SR) increases to 94.8%. Compared to the MAPPO baseline, which struggles with a 9.1% CR under steady-state wind fields, our method demonstrates robust safety awareness. Furthermore, regarding computational complexity, the average inference time per decision step (Time) for NPD3QN is recorded at 9.8 ± 0.7 ms. Although the integration of the *N*-step targets and improved PER slightly increases the computational overhead compared to the standard D3QN (8.2 ± 0.5 ms), it remains significantly more efficient than the MAPPO baseline (15.4 ± 1.2 ms). Most importantly, an inference execution time of under 10 ms demonstrates suitability for high-frequency online decision-making strictly within the context of the reported workstation hardware specifications.

Regarding the overall trajectory trends, the baseline D3QN generates paths with evident detours, reflecting its tendency to fall into local sub-optimal decisions under complex constraints. With the integration of the *N*-step update strategy, the trajectories become more rational as unnecessary detours are suppressed. This indicates that longer-term reward information assists the agent in balancing immediate actions with future gains, thereby reducing short-sighted redundancies. Similarly, the improved PER mechanism reduces detours by reinforcing the learning of high-value samples during training, which effectively prevents the algorithm from falling into local sub-optimal traps caused by early collisions. Ultimately, the multi-UAV paths generated by NPD3QN exhibit the best comprehensive performance while satisfying all feasibility constraints, validating the effective synergistic effect of the integrated modules.

The reward convergence curves, supplemented with shaded error bands representing the standard deviation across 5 independent runs, provide further insights into the training dynamics. It can be observed that in both scenarios, NPD3QN demonstrates significantly faster convergence and achieves a higher steady-state reward plateau compared to the baseline D3QN and MAPPO variants. In the more complex wind-disturbed scenario, the advantage of NPD3QN becomes even more pronounced, with the algorithm maintaining a tighter confidence interval and a smoother reward trajectory throughout the training process. This accelerated convergence and enhanced statistical stability can be attributed to the synergistic integration of *N*-step updates and the task-oriented PER mechanism, which help address the sparse reward problem and prioritize informative transitions in the simulated steady-state wind-field environment.

To further substantiate the safety justification of the modified PER mechanism and clarify its internal dynamics, a targeted ablation study was conducted on the hyperparameter pair (ρ,η). Table 7 reports the steady-state buffer composition (sampling proportions of positive, terminal collision, and near-miss transitions) alongside the corresponding path efficiency and safety metrics.

As demonstrated, using the standard PER mechanism (ρ=1.0,η=1.0) causes the buffer to be flooded with terminal collisions (42.1%), which suppresses the sampling of informative near-misses (15.3%) and results in a high Collision Rate (12.5%). By reducing the collision entry probability to ρ=0.8 and increasing the positive sampling weight to η=2.0, the buffer occupation of uninformative terminal collisions is effectively restricted to 14.2%. Concurrently, the sampling proportion of critical near-miss transitions is explicitly maximized (28.5%). This optimal configuration (ρ=0.8,η=2.0) actively improves safety (minimizing the CR to 3.6%) and path efficiency (5352.5 m) not by discarding danger signals, but by concentrating the network’s gradient updates on the most informative boundary conditions (near-misses) rather than redundant terminal failures.

### 3.6. Collision Frequency and Clearance Margin Analysis

To address potential concerns that suppressing negative experiences might degrade safety signals, and to provide a comprehensive evaluation beyond event-frequency metrics, we explicitly analyzed the continuous safety-margin statistics of the generated trajectories. As emphasized in advanced robotics, a low overall Collision Rate (CR) does not mathematically guarantee adequate physical clearance; trajectories that frequently pass dangerously close to obstacles still pose severe risks under real-world aerodynamic uncertainties. Therefore, we disaggregated the total CR into the Obstacle Collision Rate (OCR) and Inter-UAV Collision Rate (ICR), and extracted three critical clearance metrics from the successful episodes across 5 independent random seeds: the absolute Minimum Obstacle Clearance ($d_{\mathrm{obs}}^{\min}$), the 5th-Percentile Obstacle Clearance ($d_{\mathrm{obs}}^{5\%}$), and the Minimum Inter-UAV Separation ($d_{\mathrm{uav}}^{\min}$).

The detailed safety-margin statistics are summarized in Table 8. The baseline D3QN frequently yields trajectories that violate the predefined safety threshold ($d_{\mathrm{safe}}=1.5$ m), as evidenced by its minimal obstacle clearance of 0.8±0.2 m and a 5th-percentile clearance of 1.1±0.1 m. This indicates that while D3QN can occasionally reach the goal, it does so by aggressively cutting corners. The MAPPO baseline exhibits improved spatiotemporal coordination, reducing the ICR to 2.7%, but still struggles to maintain strict obstacle margins under the steady-state simulated wind field.

In contrast, the proposed NPD3QN effectively converges to a highly stable safety profile. Despite the regulated sampling density of collision data via the task-oriented PER mechanism, NPD3QN maintains an absolute minimum obstacle clearance of 1.6±0.1 m, successfully remaining above the 1.5 m safety boundary. Furthermore, its 5th-percentile clearance reaches 1.9±0.1 m, providing a robust buffer against the steady-state simulated wind-field. The simultaneous reduction in event-frequency metrics (OCR of 2.4% and ICR of 1.2%), when strictly coupled with these continuous clearance margins, suggests that explicitly defining “near-miss” spatial penalties and strategically prioritizing these safety-critical near-miss transitions during replay successfully instills rigorous safety awareness, preventing the agent from exploiting dangerously narrow airspaces.

### 3.7. Computational Cost and Complexity Analysis

To provide a comprehensive evaluation of the practical efficiency of the algorithms, both the offline training cost and the online inference complexity are explicitly reported. All models were trained and evaluated on a unified hardware and software platform, specifically a workstation equipped with an Intel Core i9-13900K CPU (Intel Corporation, Santa Clara, CA, USA), an NVIDIA GeForce RTX 3090 GPU (NVIDIA Corporation, Santa Clara, CA, USA), and 64 GB of RAM, running MATLAB (vR2023a, MathWorks, Natick, MA, USA) on a Windows 11 operating system (Microsoft Corporation, Redmond, WA, USA). Training was uniformly accelerated using the GPU to ensure fair wall-clock time comparisons.

During the offline training phase, all RL baselines were allocated a strictly comparable computational budget of $1\times 10^{6}$ environmental steps (2000 episodes × 500 maximum steps). Table 9 summarizes the precise number of trainable parameters per agent and the average wall-clock training time for each evaluated method.

As shown in Table 9, the standard DQN and D3QN-based architectures possess a relatively lightweight parameter scale due to their strictly decentralized execution structures. In contrast, MAPPO utilizes a centralized critic that processes concatenated global states, resulting in a significantly larger parameter footprint (23,131 parameters). Consequently, while the proposed NPD3QN integrates the *N*-step targets and an improved PER mechanism—which introduces a moderate increase in wall-clock training time compared to the standard D3QN due to prioritized sampling overhead—its overall training cost remains highly competitive and significantly lower than the MAPPO baseline.

Following the offline training phase, the computational burden during online path planning mainly arises from online inference. During online planning, each decision step requires one forward pass through the dueling network and one environment transition evaluation. Since the transition update involves only local motion execution, obstacle checking, and wind-field-based state update, its cost remains constant under the current grid-based implementation. Therefore, the online computational complexity is dominated by the neural network forward pass.

For a network with input dimension *d*, action dimension *A*, two shared hidden layers with widths $h_{1}$ and $h_{2}$, and value and advantage branch widths $h_{v}$ and $h_{a}$, respectively, the per-step inference complexity can be expressed as:

(44) $$O\left(dh_{1}+h_{1}h_{2}+h_{2}h_{v}+h_{2}h_{a}+h_{a}A+\left(A+1\right)\right)$$

In this study, the observation dimension is d=9, the action dimension is A=26, the shared hidden-layer widths are $h_{1}=128$ and $h_{2}=64$, and both branch widths are set to 32. Accordingly, the online decision complexity of the proposed network remains on the order of $10^{4}$ arithmetic operations per step, indicating that the inference cost is lightweight. If the planning horizon is denoted by *T*, then the online path-planning complexity for a single UAV is O(T) with a constant per-step cost, and the overall complexity for *K* UAVs is O(KT). Because the maximum number of decision steps is bounded in the implementation, the online planning complexity remains controllable for deployment.

## 4. Discussion and Conclusions

This paper investigates the multi-UAV cooperative path planning problem under the dual constraints of urban low-altitude building clusters and wind field disturbances. By integrating an *N*-step update strategy and an improved PER mechanism into the D3QN framework, and incorporating wind field information into the reward function, we propose the NPD3QN algorithm. A 3D simulation environment fusing urban architecture with 3D wind fields was developed to evaluate the efficacy of the proposed method. Simulation and ablation results confirm the validity of NPD3QN, with the primary conclusions summarized as follows:

- **High Efficiency in Deterministic Environments:** In windless environments, NPD3QN successfully plans shorter cooperative paths while achieving faster and more stable reward convergence. Compared to baseline algorithms such as A*, DQN, and D3QN, the proposed method demonstrates significantly higher learning efficiency and improved path planning quality.
- **Robustness against Wind Perturbations:** Under wind field perturbations, while the planning complexity increases for all algorithms—manifesting as increased path lengths—NPD3QN maintains an optimal or near-optimal performance. It generates trajectories with fewer detours, achieves earlier stability, and exhibits lower late-stage oscillations, underscoring its robust disturbance rejection capability and policy robustness.
- **Synergistic Algorithmic Enhancements:** Ablation studies reveal that both the *N*-step update strategy and the improved PER mechanism consistently improve path efficiency and training dynamics. Their combined application yields the highest comprehensive performance, verifying that the complementary mechanisms of reinforcing long-term returns and prioritizing critical experiences are essential for high-quality policy learning and stable convergence.
- **Kinematic-Aware Policy Learning:** By embedding wind field information into the learning framework via state awareness, dynamics drift modeling, and reward guidance, the policy effectively learns behavioral patterns such as tailwind utilization and headwind/crosswind avoidance. This enhances the reliability and safety of planning results in modeled steady-state urban wind environments, providing a viable simulated technical pathway for autonomous multi-UAV cooperative path planning in complex airspaces.

While this study provides a novel and effective solution for UAV path planning, certain limitations remain. The current model primarily focuses on a steady-state spatial vector field and abstracts the UAVs as point-mass agents with simplified kinematics. Consequently, we explicitly state that the current findings do not yet confirm real-world deployment readiness, robustness to time-varying gusts (e.g., wind shear and transient turbulence), or validity under full nonlinear UAV dynamics. Unmodeled stochastic aerodynamic effects could potentially induce trajectory deviations or degrade inter-UAV safety margins during physical execution. Future research will focus on integrating computational fluid dynamics (CFD) data, explicit geometric boundary modeling of UAV bodies, and more refined nonlinear dynamics constraints (e.g., minimum turning radii and maximum climb rates) to support more deployment-oriented simulation studies. Furthermore, by incorporating mission-specific requirements—such as target prioritization, no-fly zone constraints, and time-sensitivity for applications like reconnaissance, communication relay, and rapid response—the adaptability and scalability of NPD3QN for practical engineering applications can be further enhanced.

## Figures and Tables

**Table 1:** Comparison of related works in UAV path planning and coordination.

Study	Methodology	Multi-UAV	Environment Context	Wind-Awareness	Primary Focus
Tang et al. [11]	D3QN + PER	Single-agent	Dynamic point obstacles	Not modeled	Experience sampling efficiency
Farid et al. [12]	DQN + $L_{2}$ + PER	Single-agent	3D indoor/cluttered	Not modeled	High-dimensional state optimization
Yan et al. [24]	RRT*-APF	Multi-agent	3D urban structures	Wind interference	Hybrid path control stability
Guan et al. [25]	MAPPO	Multi-agent	Disaster/Open area	Not modeled	Cooperative deployment efficiency
**Ours**	**NPD3QN**	**Multi-agent**	**Modeled urban canyons**	**Steady-state kinematics**	**Kinematic-aware coordination**

**Table 2:** Definition of the 9-dimensional observation vector for each UAV.

Index	Component	Description	Raw Unit	Coordinate Frame	Information Source
1	$\Delta x_{g,t}^{i}$	Relative target distance in X-axis	m	Global frame	Computed from assigned target
2	$\Delta y_{g,t}^{i}$	Relative target distance in Y-axis	m	Global frame	
3	$\Delta z_{g,t}^{i}$	Relative target distance in Z-axis	m	Global frame	
4	$d_{obs,t}^{i}$	Distance to the nearest obstacle	m	Local frame	Local obstacle sensing
5	$d_{uav,t}^{i}$	Distance to the nearest neighboring UAV	m	Local frame	Local inter-UAV sensing
6	$U_{t}^{i}$	Wind velocity component in X-axis	m/s	Global frame	Local wind-field sampling
7	$V_{t}^{i}$	Wind velocity component in Y-axis	m/s	Global frame	
8	$W_{t}^{i}$	Wind velocity component in Z-axis	m/s	Global frame	
9	$v_{wind,t}^{i}$	Total wind speed magnitude	m/s	Local frame	

**Table 3:** Algorithm performance under different parameter configurations. The final optimal configuration is highlighted in bold.

Training Episodes	Parameter Configuration	Success Rate	Average Reward	Reward Std. Dev.
200	N=5,ρ=0.8,η=2.0	0.408	96.8	17.2
	N=5,ρ=0.6,η=2.0	0.373	98.6	20.1
	N=5,ρ=1.0,η=2.0	0.396	90.1	17.6
	N=5,ρ=0.8,η=3.0	0.438	90.2	21.2
	N=5,ρ=1.0,η=3.0	0.401	91.8	19.8
	N=5,ρ=0.6,η=3.0	0.400	91.6	22.9
	N=5,ρ=0.4,η=2.0	0.375	85.7	20.5
	N=5,ρ=0.4,η=3.0	0.390	89.9	25.0
	N=7,ρ=0.8,η=3.0	0.376	82.9	23.0
	N=7,ρ=1.0,η=2.0	0.348	85.1	22.2
	N=7,ρ=0.6,η=3.0	0.383	86.1	27.0
	N=7,ρ=0.4,η=3.0	0.378	86.6	26.9
	N=3,ρ=0.8,η=3.0	0.391	77.2	22.0
	N=7,ρ=0.8,η=2.0	0.382	76.3	22.7
	N=3,ρ=0.4,η=3.0	0.370	82.9	26.8
	N=3,ρ=1.0,η=3.0	0.355	79.9	25.2
	N=7,ρ=1.0,η=3.0	0.373	77.3	26.8
	N=7,ρ=0.4,η=2.0	0.358	71.4	24.0
	N=3,ρ=0.6,η=3.0	0.392	68.5	25.7
	N=7,ρ=0.6,η=2.0	0.349	68.2	23.2
	N=3,ρ=0.8,η=2.0	0.344	66.5	22.3
	N=3,ρ=0.6,η=2.0	0.343	67.1	23.2
	N=5,ρ=0.8,η=1.0	0.281	69.6	20.9
	N=3,ρ=0.4,η=2.0	0.315	68.5	23.2
	N=3,ρ=1.0,η=2.0	0.302	66.7	22.0
	N=5,ρ=0.4,η=1.0	0.270	68.2	22.7
	N=5,ρ=0.6,η=1.0	0.247	69.4	22.3
	N=5,ρ=1.0,η=1.0	0.246	57.6	22.0
	N=7,ρ=0.8,η=1.0	0.253	58.9	24.6
	N=7,ρ=0.6,η=1.0	0.241	55.9	26.1
	N=3,ρ=0.6,η=1.0	0.225	54.9	26.6
	N=3,ρ=0.4,η=1.0	0.231	52.6	27.1
	N=7,ρ=0.4,η=1.0	0.231	52.0	28.9
	N=3,ρ=0.8,η=1.0	0.206	45.4	22.8
	N=3,ρ=1.0,η=1.0	0.219	48.8	26.3
	N=7,ρ=1.0,η=1.0	0.198	49.0	25.1
600	N=5,ρ=0.8,η=3.0	0.748	169.7	21.2
	N=5,ρ=0.8,η=2.0	0.745	166.0	18.8
	N=5,ρ=0.4,η=2.0	0.673	166.5	18.3
	N=5,ρ=0.6,η=2.0	0.743	158.2	19.1
	N=5,ρ=1.0,η=2.0	0.725	153.3	19.5
	N=5,ρ=1.0,η=3.0	0.744	152.9	22.1
	N=5,ρ=0.6,η=3.0	0.708	154.0	22.6
	N=5,ρ=0.4,η=3.0	0.746	152.6	24.3
	N=7,ρ=1.0,η=2.0	0.707	140.7	22.9
	N=7,ρ=0.8,η=3.0	0.728	136.7	26.5
	N=7,ρ=0.4,η=3.0	0.680	139.5	26.4
	N=7,ρ=0.6,η=3.0	0.705	137.0	27.5
2000	N=5,ρ=0.8,η=2.0	**0.914**	**210.6**	**18.0**
	N=5,ρ=0.6,η=2.0	0.897	199.0	18.8
	N=5,ρ=0.4,η=2.0	0.863	195.7	19.7
	N=5,ρ=0.8,η=3.0	0.866	180.5	22.5

**Table 4:** Path length of A*, DQN, D3QN, MAPPO, and the proposed algorithm (no wind field).

Algorithm	Path Planning Length (m)			
	**UAV1**	**UAV2**	**UAV3**	**Total**
A*	2417.9 ± 0.0	2201.3 ± 0.0	2202.9 ± 0.0	6822.1 ± 0.0
DQN	1830.7 ± 25.4	1732.6 ± 22.1	1745.4 ± 24.8	5308.7 ± 72.3
D3QN	1712.3 ± 18.2	1612.5 ± 15.6	1677.7 ± 19.3	5002.5 ± 53.1
MAPPO	1712.3 ± 12.5	1605.4 ± 11.8	1668.2 ± 14.1	4985.9 ± 38.4
**NPD3QN (Proposed)**	**1712.3 ± 8.4**	**1598.8 ± 7.2**	**1661.1 ± 9.5**	**4972.2 ± 25.1**

**Table 5:** Path length of DQN, D3QN, MAPPO, and the proposed algorithm (with wind field).

Algorithm	Path Planning Length (m)			
	**UAV1**	**UAV2**	**UAV3**	**Total**
DQN	2289.7 ± 45.8	2154.0 ± 41.2	2239.2 ± 43.6	6682.9 ± 130.6
D3QN	2138.2 ± 32.5	1960.9 ± 28.7	1964.4 ± 30.1	6063.5 ± 91.3
MAPPO	2015.4 ± 25.1	1852.1 ± 22.4	1870.5 ± 24.8	5738.0 ± 72.3
**NPD3QN (Proposed)**	**1886.6 ± 15.2**	**1729.9 ± 12.8**	**1736.0 ± 14.5**	**5352.5 ± 42.5**

**Table 6:** Comprehensive performance and ablation analysis with multiple metrics (With wind field). Here, SR denotes Success Rate, CR denotes Collision Rate, and Time represents the average inference time per decision step, measured on the reported workstation hardware.

Algorithm	Path Length (m)				SR (%)	CR (%)	Time (ms)
	**UAV1**	**UAV2**	**UAV3**	**Total**			
D3QN (Baseline)	2138.2 ± 32.5	1960.9 ± 28.7	1964.4 ± 30.1	6063.5 ± 91.3	78.4 ± 3.2	12.5 ± 1.8	8.2 ± 0.5
D3QN with *N*-steps	2050.4 ± 22.4	1926.0 ± 20.1	1847.2 ± 21.5	5823.6 ± 65.4	84.2 ± 2.5	8.4 ± 1.2	9.4 ± 0.6
D3QN with PER	1952.7 ± 19.6	1831.7 ± 17.4	1642.6 ± 18.2	5427.0 ± 52.8	88.6 ± 1.8	6.8 ± 0.9	8.5 ± 0.5
MAPPO	2015.4 ± 25.1	1852.1 ± 22.4	1870.5 ± 24.8	5738.0 ± 72.3	82.5 ± 2.1	9.1 ± 1.5	15.4 ± 1.2
**NPD3QN**	**1886.6 ± 15.2**	**1729.9 ± 12.8**	**1736.0 ± 14.5**	**5352.5 ± 42.5**	**94.8 ± 1.2**	**3.6 ± 0.5**	**9.8 ± 0.7**

**Table 7:** Buffer composition and performance metrics under varying ρ and η configurations.

ρ (Entry Prob.)	η (Weight)	Buffer Sampling Proportion (%)			Total Path Length (m) ↓	Collision Rate (%) ↓
		**Collision $\left(I_{\mathrm{pos}}=0\right)$**	**Near-Miss $\left(I_{\mathrm{pos}}=1\right)$**	**Other Positive**		
1.0 (Standard)	1.0	42.1	15.3	42.6	6063.5 ± 91.3	12.5 ± 1.8
0.8	1.0	28.6	18.2	53.2	5712.4 ± 68.2	9.2 ± 1.4
1.0	2.0	31.4	24.5	44.1	5645.8 ± 55.6	7.8 ± 1.1
**0.8 (Ours)**	**2.0 (Ours)**	**14.2**	**28.5**	**57.3**	**5352.5 ± 42.5**	**3.6 ± 0.5**

**Table 8:** Comprehensive safety-margin and collision statistics across 5 random seeds (with wind field).

Algorithm	OCR (%) ↓	ICR (%) ↓	$d_{\mathrm{obs}}^{\min}$ (m) ↑	$d_{\mathrm{obs}}^{5\%}$ (m) ↑	$d_{\mathrm{uav}}^{\min}$ (m) ↑
D3QN (Baseline)	8.2 ± 1.1	4.3 ± 0.7	0.8 ± 0.2	1.1 ± 0.1	1.2 ± 0.3
MAPPO	6.4 ± 0.9	2.7 ± 0.6	1.2 ± 0.1	1.4 ± 0.1	1.5 ± 0.2
**NPD3QN (Ours)**	**2.4 ± 0.4**	**1.2 ± 0.3**	**1.6 ± 0.1**	**1.9 ± 0.1**	**1.8 ± 0.1**

**Table 9:** Comparison of offline training computational costs across 5 random seeds.

Algorithm	Trainable Parameters	Total Training Steps	Hardware Acceleration	Wall-Clock Time (Hours)
DQN	11,226	$1\times 10^{6}$	GPU	2.5 ± 0.2
D3QN (Baseline)	14,587	$1\times 10^{6}$	GPU	3.1 ± 0.2
MAPPO	23,131	$1\times 10^{6}$	GPU	5.8 ± 0.4
**NPD3QN (Ours)**	**14,587**	** $1\times 10^{6}$ **	**GPU**	**3.6 ± 0.3**

## Data Availability

The source code for the NPD3QN algorithm, simulation environment configurations, and training scripts are publicly available on GitHub at https://github.com/winnieks7/NPD3QN (accessed on 5 May 2026). Any further inquiries or requests for additional raw data can be directed to the corresponding author.
